# Supplementary material for: Improving malaria case management with artemisinin-based combination therapies and malaria rapid diagnostic tests in private medicine retail outlets in sub-Saharan Africa: A systematic review
Source: PLoS One. 2024 Jul 29;19(7):e0286718. doi: 10.1371/journal.pone.0286718 (PMC11285950; doi:10.1371/journal.pone.0286718)
Supplement: S1 Table — (DOCX) [file pone.0286718.s001.docx]

## S1 Table. Search strategy

Detailed search strategies are presented for Medline, Embase, Global Health, and EconLit. To maximise the efficiency of the search, terms were strategically combined, such that papers with terms related to “Diagnosis & treatment” also had to include terms related to “Malaria & fever” to be eligible for abstract screening. However, papers with terms related to “Antimalarials” did not have to contain terms related to “Malaria & fever” to be eligible for screening. The numbers of studies identified are based on searches conducted on March 10^th^ 2023. All databases were accessed through the LSHTM portal and the resource selection was set to “Ovid MEDLINE (2) and Epub Ahead of Print, In-Process, In-Data-Review & Other Non-Indexed Citations, Daily and Versions® for Medline Ovid 1949 to Mar 9, 2023, Embase 1974 to Mar 9 2023, Global Health 1910 to 2023 week 09, and EconLit 1886 to Feb, 2023.

| Medline OVID | | | |
| --- | --- | --- | --- |
| Search Number | Concept | Search Term | Results |
| 1 | Malaria & fever | (malaria or fever or fevers or febrile).mp. or exp fever/ or exp malaria/ | 373719 |
| 2 | Private medicine retailers | (Private or retail* or drug seller* or drug vendor* or drug store* or pharmacy or pharmacies or informal provider* or patent medicine vendor* or shop* or PMV or PMR or PPMV or drug dispensing outlet*).mp. | 227730 |
| 3 | Diagnosis & treatment | (test or tests or diagnostic* or RDT or ACT or treatment).mp. | 10059611 |
| 4 | Antimalarials | (artemisinin* or antimalarial* or anti-malarial*).mp. or exp antimalarials/ | 99989 |
| 5 | Sub-Saharan Africa | Exp "Africa south of the Sahara"/ or ("Africa South of the Sahara" or sub-Saharan Africa or subSaharan Africa).ti,ab. or Central Africa.ti,ab. or Eastern Africa.ti,ab. or Southern Africa.ti,ab. or Western Africa.ti,ab. or Seychelles/ or Seychelles.ti,ab. or Benin/ or (Benin or Dahomey).ti,ab. or Burkina Faso/ or (Burkina Faso or Burkina Fasso or Upper Volta).ti,ab. or Burundi/ or (Burundi or Ruanda-Urundi).ti,ab. or Central African Republic/ or (Central African Republic or Ubangi-Shari).ti,ab. or Chad/ or Chad.ti,ab. or Democratic Republic Congo/ or (((Democratic Republic or DR) adj2 Congo) or Congo-Kinshasa or Belgian Congo or Zaire or Congo Free State).ti,ab. or Eritrea/ or Eritrea.ti,ab. or Ethiopia/ or (Ethiopia or Abyssinia).ti,ab. or Gambia/ or Gambia.ti,ab. or Guinea/ or (Guinea not (New Guinea or Guinea Pig* or Guinea Fowl or Guinea-Bissau or Portuguese Guinea or Equatorial Guinea)).ti,ab. or Guinea-Bissau/ or (Guinea-Bissau or Portuguese Guinea).ti,ab. or Liberia/ or Liberia.ti,ab. or Madagascar/ or (Madagascar or Malagasy Republic).ti,ab. or Malawi/ or (Malawi or Nyasaland).ti,ab. or Mali/ or Mali.ti,ab. or Mozambique/ or (Mozambique or Mocambique or Portuguese East Africa).ti,ab. or Niger/ or (Niger not (Aspergillus or Peptococcus or Schizothorax or Cruciferae or Gobius or Lasius or Agelastes or Melanosuchus or radish or Parastromateus or Orius or Apergillus or Parastromateus or Stomoxys)).ti,ab. or Rwanda/ or (Rwanda or Ruanda).ti,ab. or Sierra Leone/ or (Sierra Leone or Salone).ti,ab. or Somalia/ or (Somalia or Somaliland).ti,ab. or south sudan/ or South Sudan.ti,ab. or Tanzania/ or (Tanzania or Tanganyika or Zanzibar).ti,ab. or Togo/ or (Togo or Togolese Republic or Togoland).ti,ab. or Uganda/ or Uganda.ti,ab. or Angola/ or Angola.ti,ab. or Cameroon/ or (Cameroon or Kamerun or Cameroun).ti,ab. or Cape Verde/ or (Cape Verde or Cabo Verde).ti,ab. or Comoros/ or (Comoros or Glorioso Islands or Mayotte).ti,ab. or Congo/ or (Congo not ((Democratic Republic adj3 Congo) or congo red or crimean-congo)).ti,ab. or Cote d'Ivoire/ or (Cote d'Ivoire or Cote dIvoire or Ivory Coast).ti,ab. or eswatini/ or (eSwatini or Swaziland).ti,ab. or Ghana/ or (Ghana or Gold Coast).ti,ab. or Kenya/ or (Kenya or East Africa Protectorate).ti,ab. or Lesotho/ or (Lesotho or Basutoland).ti,ab. or Mauritania/ or Mauritania.ti,ab. or Nigeria/ or Nigeria.ti,ab. or "sao tome and principe"/ or (Sao Tome adj2 Principe).ti,ab. or Senegal/ or Senegal.ti,ab.or Sudan/ or (Sudan not South Sudan).ti,ab. or Zambia/ or (Zambia or Northern Rhodesia).ti,ab. or Zimbabwe/ or (Zimbabwe or Southern Rhodesia).ti,ab. or Botswana/ or (Botswana or Bechuanaland or Kalahari).ti,ab. or Equatorial Guinea/ or (Equatorial Guinea or Spanish Guinea).ti,ab. or Gabon/ or (Gabon or Gabonese Republic).ti,ab. or Mauritius/ or (Mauritius or Agalega Islands).ti,ab. or Namibia/ or (Namibia or German South West Africa).ti,ab. or South Africa/ or (South Africa or Cape Colony or British Bechuanaland or Boer Republics or Zululand or Transvaal or Natalia Republic or Orange Free State).ti,ab. | 342934 |
| 1 AND 3 | 6 |  | 150213 |
| 6 OR 4 | 7 |  | 232663 |
| 7 and 2 and 5 | 8 |  | 797 |
| 8 + time limit (2006-10 March 2023) + language limit (English or French) | 9 |  | 688 |

| Embase OVID | | | |
| --- | --- | --- | --- |
| Search Number | Concept | Search Term | Results |
| 1 | Malaria & fever | (malaria or fever or fevers or febrile).mp. or exp fever/ or exp malaria/ | 667427 |
| 2 | Private medicine retailers | (Private or retail* or drug seller* or drug vendor* or drug store* or pharmacy or pharmacies or informal provider* or patent medicine vendor* or shop* or PMV or PMR or PPMV or drug dispensing outlet*).mp. | 363906 |
| 3 | Diagnosis & treatment | (test or tests or diagnostic* or RDT or ACT or treatment).mp. | 12843244 |
| 4 | Antimalarials | (artemisinin* or antimalarial* or anti-malarial*).mp. or exp antimalarials/ | 181375 |
| 5 | Sub-Saharan Africa | Exp "Africa south of the Sahara"/ or ("Africa South of the Sahara" or sub-Saharan Africa or subSaharan Africa).ti,ab. or Central Africa.ti,ab. or Eastern Africa.ti,ab. or Southern Africa.ti,ab. or Western Africa.ti,ab. or Seychelles/ or Seychelles.ti,ab. or Benin/ or (Benin or Dahomey).ti,ab. or Burkina Faso/ or (Burkina Faso or Burkina Fasso or Upper Volta).ti,ab. or Burundi/ or (Burundi or Ruanda-Urundi).ti,ab. or Central African Republic/ or (Central African Republic or Ubangi-Shari).ti,ab. or Chad/ or Chad.ti,ab. or Democratic Republic Congo/ or (((Democratic Republic or DR) adj2 Congo) or Congo-Kinshasa or Belgian Congo or Zaire or Congo Free State).ti,ab. or Eritrea/ or Eritrea.ti,ab. or Ethiopia/ or (Ethiopia or Abyssinia).ti,ab. or Gambia/ or Gambia.ti,ab. or Guinea/ or (Guinea not (New Guinea or Guinea Pig* or Guinea Fowl or Guinea-Bissau or Portuguese Guinea or Equatorial Guinea)).ti,ab. or Guinea-Bissau/ or (Guinea-Bissau or Portuguese Guinea).ti,ab. or Liberia/ or Liberia.ti,ab. or Madagascar/ or (Madagascar or Malagasy Republic).ti,ab. or Malawi/ or (Malawi or Nyasaland).ti,ab. or Mali/ or Mali.ti,ab. or Mozambique/ or (Mozambique or Mocambique or Portuguese East Africa).ti,ab. or Niger/ or (Niger not (Aspergillus or Peptococcus or Schizothorax or Cruciferae or Gobius or Lasius or Agelastes or Melanosuchus or radish or Parastromateus or Orius or Apergillus or Parastromateus or Stomoxys)).ti,ab. or Rwanda/ or (Rwanda or Ruanda).ti,ab. or Sierra Leone/ or (Sierra Leone or Salone).ti,ab. or Somalia/ or (Somalia or Somaliland).ti,ab. or south sudan/ or South Sudan.ti,ab. or Tanzania/ or (Tanzania or Tanganyika or Zanzibar).ti,ab. or Togo/ or (Togo or Togolese Republic or Togoland).ti,ab. or Uganda/ or Uganda.ti,ab. or Angola/ or Angola.ti,ab. or Cameroon/ or (Cameroon or Kamerun or Cameroun).ti,ab. or Cape Verde/ or (Cape Verde or Cabo Verde).ti,ab. or Comoros/ or (Comoros or Glorioso Islands or Mayotte).ti,ab. or Congo/ or (Congo not ((Democratic Republic adj3 Congo) or congo red or crimean-congo)).ti,ab. or Cote d'Ivoire/ or (Cote d'Ivoire or Cote dIvoire or Ivory Coast).ti,ab. or eswatini/ or (eSwatini or Swaziland).ti,ab. or Ghana/ or (Ghana or Gold Coast).ti,ab. or Kenya/ or (Kenya or East Africa Protectorate).ti,ab. or Lesotho/ or (Lesotho or Basutoland).ti,ab. or Mauritania/ or Mauritania.ti,ab. or Nigeria/ or Nigeria.ti,ab. or "sao tome and principe"/ or (Sao Tome adj2 Principe).ti,ab. or Senegal/ or Senegal.ti,ab.or Sudan/ or (Sudan not South Sudan).ti,ab. or Zambia/ or (Zambia or Northern Rhodesia).ti,ab. or Zimbabwe/ or (Zimbabwe or Southern Rhodesia).ti,ab. or Botswana/ or (Botswana or Bechuanaland or Kalahari).ti,ab. or Equatorial Guinea/ or (Equatorial Guinea or Spanish Guinea).ti,ab. or Gabon/ or (Gabon or Gabonese Republic).ti,ab. or Mauritius/ or (Mauritius or Agalega Islands).ti,ab. or Namibia/ or (Namibia or German South West Africa).ti,ab. or South Africa/ or (South Africa or Cape Colony or British Bechuanaland or Boer Republics or Zululand or Transvaal or Natalia Republic or Orange Free State).ti,ab. | 376493 |
| 1 AND 3 | 6 |  | 350719 |
| 6 OR 4 | 7 |  | 501591 |
| 7 and 2 and 5 | 8 |  | 1358 |
| 8 + time limit (2006-10 March 2023) + language limit (English or French) | 9 |  | 1240 |

| Global Health OVID | | | |
| --- | --- | --- | --- |
| Search Number | Concept | Search Term | Results |
| 1 | Malaria & fever | (malaria or fever or fevers or febrile).mp. or exp fever/ or exp malaria/ | 252924 |
| 2 | Private medicine retailers | (Private or retail* or drug seller* or drug vendor* or drug store*or pharmacy or pharmacies or informal provider* or patent medicine vendor* or shop* or PMV or PMR or PPMV or drug dispensing outlet*).mp. | 65699 |
| 3 | Diagnosis & treatment | (test or tests or diagnostic* or RDT or ACT or treatment).mp. | 1565000 |
| 4 | Antimalarials | (artemisinin* or antimalarial* or anti-malarial*).mp. or exp antimalarials/ | 54728 |
| 5 | Sub-Saharan Africa | Exp "Africa south of the Sahara"/ or ("Africa South of the Sahara" or sub-Saharan Africa or subSaharan Africa).ti,ab. or Central Africa.ti,ab. or Eastern Africa.ti,ab. or Southern Africa.ti,ab. or Western Africa.ti,ab. or Seychelles/ or Seychelles.ti,ab. or Benin/ or (Benin or Dahomey).ti,ab. or Burkina Faso/ or (Burkina Faso or Burkina Fasso or Upper Volta).ti,ab. or Burundi/ or (Burundi or Ruanda-Urundi).ti,ab. or Central African Republic/ or (Central African Republic or Ubangi-Shari).ti,ab. or Chad/ or Chad.ti,ab. or Democratic Republic Congo/ or (((Democratic Republic or DR) adj2 Congo) or Congo-Kinshasa or Belgian Congo or Zaire or Congo Free State).ti,ab. or Eritrea/ or Eritrea.ti,ab. or Ethiopia/ or (Ethiopia or Abyssinia).ti,ab. or Gambia/ or Gambia.ti,ab. or Guinea/ or (Guinea not (New Guinea or Guinea Pig* or Guinea Fowl or Guinea-Bissau or Portuguese Guinea or Equatorial Guinea)).ti,ab. or Guinea-Bissau/ or (Guinea-Bissau or Portuguese Guinea).ti,ab. or Liberia/ or Liberia.ti,ab. or Madagascar/ or (Madagascar or Malagasy Republic).ti,ab. or Malawi/ or (Malawi or Nyasaland).ti,ab. or Mali/ or Mali.ti,ab. or Mozambique/ or (Mozambique or Mocambique or Portuguese East Africa).ti,ab. or Niger/ or (Niger not (Aspergillus or Peptococcus or Schizothorax or Cruciferae or Gobius or Lasius or Agelastes or Melanosuchus or radish or Parastromateus or Orius or Apergillus or Parastromateus or Stomoxys)).ti,ab. or Rwanda/ or (Rwanda or Ruanda).ti,ab. or Sierra Leone/ or (Sierra Leone or Salone).ti,ab. or Somalia/ or (Somalia or Somaliland).ti,ab. or south sudan/ or South Sudan.ti,ab. or Tanzania/ or (Tanzania or Tanganyika or Zanzibar).ti,ab. or Togo/ or (Togo or Togolese Republic or Togoland).ti,ab. or Uganda/ or Uganda.ti,ab. or Angola/ or Angola.ti,ab. or Cameroon/ or (Cameroon or Kamerun or Cameroun).ti,ab. or Cape Verde/ or (Cape Verde or Cabo Verde).ti,ab. or Comoros/ or (Comoros or Glorioso Islands or Mayotte).ti,ab. or Congo/ or (Congo not ((Democratic Republic adj3 Congo) or congo red or crimean-congo)).ti,ab. or Cote d'Ivoire/ or (Cote d'Ivoire or Cote dIvoire or Ivory Coast).ti,ab. or eswatini/ or (eSwatini or Swaziland).ti,ab. or Ghana/ or (Ghana or Gold Coast).ti,ab. or Kenya/ or (Kenya or East Africa Protectorate).ti,ab. or Lesotho/ or (Lesotho or Basutoland).ti,ab. or Mauritania/ or Mauritania.ti,ab. or Nigeria/ or Nigeria.ti,ab. or "sao tome and principe"/ or (Sao Tome adj2 Principe).ti,ab. or Senegal/ or Senegal.ti,ab.or Sudan/ or (Sudan not South Sudan).ti,ab. or Zambia/ or (Zambia or Northern Rhodesia).ti,ab. or Zimbabwe/ or (Zimbabwe or Southern Rhodesia).ti,ab. or Botswana/ or (Botswana or Bechuanaland or Kalahari).ti,ab. or Equatorial Guinea/ or (Equatorial Guinea or Spanish Guinea).ti,ab. or Gabon/ or (Gabon or Gabonese Republic).ti,ab. or Mauritius/ or (Mauritius or Agalega Islands).ti,ab. or Namibia/ or (Namibia or German South West Africa).ti,ab. or South Africa/ or (South Africa or Cape Colony or British Bechuanaland or Boer Republics or Zululand or Transvaal or Natalia Republic or Orange Free State).ti,ab. | 272687 |
| 1 AND 3 | 6 |  | 104213 |
| 6 OR 4 | 7 |  | 136947 |
| 7 and 2 and 5 | 8 |  | 830 |
| 8 + time limit (2006-10 March 2023) + language limit (English or French) | 9 |  | 640 |

| Econlit OVID | | | |
| --- | --- | --- | --- |
| Search Number | Concept | Search Term | Results |
| 1 | Malaria & fever | (malaria or fever or fevers or febrile).mp. or (malaria or fever or fevers or febrile).m_titl. | 837 |
| 2 | Private medicine retailers | (Private or retail* or drug seller* or drug vendor* or drug store* or pharmacy or pharmacies or informal provider* or patent medicine vendor* or shop* or PMV or PMR or PPMV or drug dispensing outlet*).mp. | 138146 |
| 3 | Diagnosis & treatment | (test or tests or diagnostic* or RDT or ACT or treatment).mp. | 154364 |
| 4 | Antimalarials | (artemisinin* or antimalarial* or anti-malarial*).mp. or antimalarial*.m_titl. | 73 |
| 5 | Sub-Saharan Africa | "Africa south of the Sahara".mp. or ("Africa South of the Sahara" or sub-Saharan Africa or subSaharan Africa).mp. or Central Africa.mp or Eastern Africa.mp or Southern Africa.mp. or Western Africa.mp or Seychelles.mp or (Benin or Dahomey).mp or (Burkina Faso or Burkina Fasso or Upper Volta).mp or (Burundi or Ruanda-Urundi).mp or (Central African Republic or Ubangi-Shari).mp or Chad.mp or (((Democratic Republic or DR) adj2 Congo) or Congo-Kinshasa or Belgian Congo or Zaire or Congo Free State).mp or Eritrea.mp or (Ethiopia or Abyssinia).mp or Gambia.mp or (Guinea not (New Guinea or Guinea Pig* or Guinea Fowl or Guinea-Bissau or Portuguese Guinea or Equatorial Guinea)).mp or (Guinea-Bissau or Portuguese Guinea).mp or Liberia.mp or (Madagascar or Malagasy Republic).mp or (Malawi or Nyasaland).mp or Mali.mp or (Mozambique or Mocambique or Portuguese East Africa).mp or (Niger not (Aspergillus or Peptococcus or Schizothorax or Cruciferae or Gobius or Lasius or Agelastes or Melanosuchus or radish or Parastromateus or Orius or Apergillus or Parastromateus or Stomoxys)).mp or (Rwanda or Ruanda).mp or (Sierra Leone or Salone).mp or (Somalia or Somaliland).mp or South Sudan.mp or (Tanzania or Tanganyika or Zanzibar).mp or (Togo or Togolese Republic or Togoland).mp or Uganda.mp or Angola.mp or (Cameroon or Kamerun or Cameroun).mp or (Cape Verde or Cabo Verde).mp or (Comoros or Glorioso Islands or Mayotte).mp or (Congo not ((Democratic Republic adj3 Congo) or congo red or crimean-congo)).mp or (Cote d'Ivoire or Cote dIvoire or Ivory Coast).mp or (eSwatini or Swaziland).mp or (Ghana or Gold Coast).mp or (Kenya or East Africa Protectorate).mp or (Lesotho or Basutoland).mp or Mauritania.mp or Nigeria.mp or (Sao Tome adj2 Principe).mp or Senegal.mp or (Sudan not South Sudan).mp or (Zambia or Northern Rhodesia).mp or (Zimbabwe or Southern Rhodesia).mp or (Botswana or Bechuanaland or Kalahari).mp or (Equatorial Guinea or Spanish Guinea).mp or (Gabon or Gabonese Republic).mp or (Mauritius or Agalega Islands).mp or (Namibia or German South West Africa).mp or (South Africa or Cape Colony or British Bechuanaland or Boer Republics or Zululand or Transvaal or Natalia Republic or Orange Free State).mp or (Mauritius or Agalega Islands).mp | 52621 |
| 1 AND 3 | 6 |  | 211 |
| 6 OR 4 | 7 |  | 242 |
| 7 and 2 and 5 | 8 |  | 31 |
| 8 + time limit (2006-10 March 2023) + language limit (English or French) | 9 |  | 30 |
