# Supplementary material for: Improving malaria case management with artemisinin-based combination therapies and malaria rapid diagnostic tests in private medicine retail outlets in sub-Saharan Africa: A systematic review
Source: PLoS One. 2024 Jul 29;19(7):e0286718. doi: 10.1371/journal.pone.0286718 (PMC11285950; doi:10.1371/journal.pone.0286718)
Supplement: S2 Table — (DOCX) [file pone.0286718.s002.docx]

## S2 Table. Quality assessment checklist

We created a bespoke quality assessment checklist which was appropriate for the wide range of study designs included, and efficient to use, drawing primarily on Downs and Black, with some additions/modifications from the Effective Public Health Practice Project (EPHPP) quality assessment tool for quantitative studies, Drummond’s checklist for assessing economic evaluations, and the Newcastle-Ottawa Scale for assessing the quality of nonrandomised studies in meta-analyses. Studies with a contemporaneous control were scored out of 12, and those without, out of 9.

| **Checklist items** | **Instructions/Clarification provided by Downs and Black** | **Description of modifications or further instructions** | **Source** |
| --- | --- | --- | --- |
| **Reporting** |  |  |  |
| 1) Are the characteristics of the PMR included in the study clearly described? | In cohort studies and trials, inclusion and/or exclusion criteria should be given. In case-control studies, a case-definition and the source for controls should be given. |  | Downs and Black |
| 2) Are the interventions of interest clearly described? | Treatments and placebo (where relevant) that are to be compared should be clearly described. | ‘Yes' if following aspects of intervention described (or referenced) where relevant:   - Target outlet types and number - Intervention type - Intervention components - Dates/duration - Subsidy structure - Product distribution mechanism - Duration of training - Style of training (face-to-face or online) - Type of accreditation - Type of communications - Disposal of waste - Type of digital solutions - Surveillance type - Contribution to intervention of surveillance | Downs and Black |
| 3) Have the characteristics of PMR lost to follow-up been described? | This should be answered yes where there were no losses to follow-up or where losses to follow-up were so small that findings would be unaffected by their inclusion. This should be answered ‘no’ where a study does not report the number of patients lost to follow-up. | Considered 'yes' if levels of unit/item non- response described | Downs and Black |
| **External validity** |  |  |  |
| 4) Were those subjects who participated representative of the target population? |  |  | Downs and Black |
| 5) Does the implementation of the intervention reflect what would happen in regular practice? |  | No, if the intervention contained elements that were unlikely to occur outside of a trial setting (e.g. RDTs provided to patients in PMRs for free). | Modified from Drummond |
| **Internal validity** |  |  |  |
| 6) Were the statistical tests used to assess the main outcomes appropriate? | The statistical techniques used must be appropriate to the data. For example, nonparametric methods should be used for small sample sizes. Where little statistical analysis has been undertaken but where there is no evidence of bias, the question should be answered yes. If the distribution of the data (normal or not) is not described it must be assumed that the estimates used were appropriate and the question should be answered yes. | Including was the analysis adjusted for clustering? | Downs and Black |
| 7) Was compliance with the intervention/s reliable? | Where there was non-compliance with the allocated treatment or where there was contamination of one group, the question should be answered no. For studies where the effect of any misclassification was likely to bias any association to the null, the question should be answered yes. |  | Downs and Black |
| 8) Was the length of follow-up adequate? | If the length of follow-up was more than 5 months, score yes. |  | Modified from Newcastle-Ottawa Quality Assessment Scale |
| 9) Were there important differences between groups prior to the intervention? | For example, subjects for all comparison groups should be selected from a similar geographical area. The question should be answered unable to determine for cohort and case control studies where there is no information concerning the source of subjects included in the study. | n/a for studies without a contemporaneous control | From EPHPP ‘quality assessment tool for quantitative studies’ |
| 10) Were study subjects randomised to intervention groups? | Were study subjects randomised to intervention groups? | n/a for studies without a contemporaneous control | Downs and Black |
| 11) Was there adequate adjustment for confounding in the analyses from which the main findings were drawn? | This question should be answered no for trials if: the main conclusions of the study were based on analyses of treatment rather than intention to treat; the distribution of known confounders in the different treatment groups was not described; or the distribution of known confounders differed between the treatment groups but was not taken into account in the analyses. In nonrandomized studies if the effect of the main confounders was not investigated or confounding was demonstrated but no adjustment was made in the final analyses | n/a for studies without a contemporaneous control | Downs and Black |
| 12) Did the study have sufficient power to detect a clinically important effect where the probability value for a difference being due to chance is less than 5%? | Sample sizes have been calculated to detect a difference of x% and y%. |  | Downs and Black |

References:

- Downs SH, Black N. The feasibility of creating a checklist for the assessment of the methodological quality both of randomised and non-randomised studies of health care interventions. Journal of epidemiology and community health. 1998;52(6):377-84.
- Drummond M, McGuire A. Economic Evaluation in Health Care - Merging theory with practice. OUP 2021
- The Effective Public Health Practice Project (EPHPP) Quality Assessment tool for Quantitative Studies <https://merst.ca/ephpp/>

The Newcastle-Ottawa Scale (NOS) for assessing the quality of nonrandomised studies in meta-analyses <http://www.ohri.ca/programs/clinical_epidemiology/oxford.asp>
