# Supplementary material for: Improving malaria case management with artemisinin-based combination therapies and malaria rapid diagnostic tests in private medicine retail outlets in sub-Saharan Africa: A systematic review
Source: PLoS One. 2024 Jul 29;19(7):e0286718. doi: 10.1371/journal.pone.0286718 (PMC11285950; doi:10.1371/journal.pone.0286718)
Supplement: S3 Table — (DOCX) [file pone.0286718.s003.docx]

S3 Table. Study and intervention characteristics

| Study Design | | Study Setting | | Intervention Components | | | | | |  |
| --- | --- | --- | --- | --- | --- | --- | --- | --- | --- | --- |
| First author,  Yr published;  Country;  Comparison | Study Type;  Date & duration of intervention | Urban/ rural;  Malaria transmission^1^ | Type & no. of outlets in intervention arm(s) | PMR training | PMR supervision | Mass comms. | Individual comms. | Product distribution | Product subsidies^2^ |  |
| 1. Introducing and enhancing ACT use (without diagnostics) | | | | | | | | | | |
| 1.1 Sub-national ACT subsidy programmes | | | | | | | | | | |
| Kangwana 2011, Kangwana 2013  Kenya  Subsidised paediatric ACTs v. no subsidy | Cluster RCT  2008-09  8 months | Rural  High / moderate | Registered or unregistered pharmacies (similar to drug shops elsewhere) 61  General stores 164  Must be operating for 6 months, selling antimalarials or antipyretics in past year, and attend training | 1 day | Quarterly | Community sensitisation through leader workshops, community events, group discussions, community based- organisation outreaches, and distribution of promotional materials, etc | - | Project staff delivered ACTs directly to trained outlets monthly | Retail purchase price: 2008 USD 0.10  Recommended retail selling price (RRP): 2008 USD 0.25 (printed on pack) |  |
| Lussiana 2016  Angola  Subsidised paediatric ACTs v. no subsidy | Pre-post without control  2009-20135  3 years | Urban and rural  Low | Pharmacies 165  Must be licensed, attend intervention training, and pass a test | Annual training; duration not stated | Monthly | Community theatre, printed materials, radio broadcasts | - | Direct distribution to pharmacies by project staff based on orders^6^ | Retail purchase price: 2013 USD 0.52  RRP: 2013 USD 0.78 (printed on pack) |  |
| Sabot 2009  Tanzania  Subsidised ACTs without RRP v. subsidised ACTs with RRP v. no subsidy | Pre-post with control  2007-2008  15 months | Rural  Low to moderate / moderate | All drug shops in the intervention areas  No RRP arm: 83  RRP arm: 68 | 1 day | - | Radio advertisements, wall paintings, themed cultural shows | - | ACTs sold by project to 1 private wholesaler in the capital. Wholesaler sold ACTs to drug shops in the intervention districts through normal distribution chain. | ACTs sold to wholesalers for an average of USD 0.11. There was no set price for the wholesaler to sell to drug shops.   RRP arm only: RRP of USD 0.25, 0.50, 0.75, 1.00 (printed on pack) |  |
| Talisuna 2012  Uganda  Subsidised ACTs v. no subsidy | Pre-post with control  2008-2010  2 years | Rural  High | Drug shops (number in intervention arm not stated)  Must be licensed | Initial and refresher training; duration not stated | - | Community events, radio spots, talk shows, posters, other point of sale advertising, songs, community events | - | Two models for direct distribution to shops were used (with costs of weekly distribution either covered by intervention funder or distributor) | RRP: USD 0.10, 0.20, 0.30, 0.40 (printed on pack) |  |
| 1.2 National ACT subsidy programmes | | | | | | | | | | |
| ACTwatch 2017  Kenya, Madagascar, Nigeria, Tanzania, Uganda  CPM v. AMFm v. no subsidy | Pre-post without control  2013-2015^7^  3 years | Urban and rural  High/moderate: Nigeria, Uganda  Low to moderate: Tanzania  Low / low to moderate: Kenya, Madagascar | All private for-profit outlets within participating countries could purchase subsidised ACTs | Dates of training during CPM period:  Kenya: 2013-2014  Madagascar: Q1-Q3 of 2015  Nigeria: 2014  Tanzania: None  Uganda: Q4 2015  Details of training not reported. | - | Duration of activities during CPM period:  Kenya: 2013-mid 2015  Madagascar: Q1-Q3 of 2015  Nigeria: 2014  Tanzania: Q4 2014- Q1 2015  Uganda: None  Details of comms not reported. | - | Eligible importers place orders to the country’s Global Fund principal recipient for approval. Global Fund then authorises co-payment to manufacturer.  After delivery to importer, ACTs distributed through normal distribution chain. | Co-payment levels set by each country  Kenya: 95% subsidy to mid-2013, then 70%  Madagascar: 95%  Nigeria: 95% to mid-2013, then 85%  Tanzania: 95% to 2014, then 80% (adult) and 90% (paed.), then 2015 onwards 75% (adult) and 85% (paed.)  Uganda: 95% to 2014, then 50% (adult) and 75% (paed.), then mid-2015 onwards 70%  RRPs not stated |  |
| Fink 2013  Uganda  AMFm v. no ACT subsidy | Pre-post without control  2011-2012  1 year | Rural  High | All private for-profit outlets within country could purchase subsidised ACTs.  This study focuses on 6 districts only | See IE Team 2012, Tougher 2012 | See IE Team 2012, Tougher 2012 | See IE Team 2012, Tougher 2012 | - | See IE Team 2012, Tougher 2012 | See IE Team 2012, Tougher 2012  Subsidised RDTs available in some shops (see Cohen 2015) |  |
| Fiore 2018  Ghana, Nigeria, & Uganda  AMFm/CPM v. comparator countries with no AMFm/CPM | Pre-post with control  2006-2016  (Last data sets 2016 (Ghana and Nigeria) and 2015 (Uganda))  >4 years | Urban and rural  High: Ghana  High/moderate: Nigeria, Uganda | All private for-profit outlets within country could purchase subsidised ACTs. | See ACTwatch 2017 for description of CPM; see IE Team 2012, Tougher 2012 for description of AMFm | See ACTwatch 2017 for description of CPM; see IE Team 2012, Tougher 2012 for description of AMFm | See ACTwatch 2017 for description of CPM; see IE Team 2012, Tougher 2012 for description of AMFm | - | See ACTwatch 2017 for description of CPM; see IE Team 2012, Tougher 2012 for description of AMFm | See ACTwatch 2017 for description of CPM; see IE Team 2012, Tougher 2012 for description of AMFm |  |
| IE Team 2012, Tougher 2012  Ghana, Kenya, Madagascar, Niger, Nigeria, Tanzania (mainland & Zanzibar), Uganda  AMFm v. no ACT subsidy | Pre-post without control  2010-2012  9 -18 months | Urban and rural  High: Ghana, Niger, Nigeria, Uganda  Low to moderate: Tanzania  Low/low to moderate: Kenya, Madagascar | All private for-profit outlets within country could purchase subsidised ACTs. | Training of selected PMR in Ghana, Kenya, Madagascar, Tanzania (Mainland and Zanzibar)  No training in PMR in Niger, Nigeria and Uganda | Supervision visits to selected PMR reported in Ghana, Kenya and Niger | Comms activities implemented at scale except Uganda^8^  Included a range of activities such as mass media advertisements and community promotion. | - | Registered wholesalers place orders with participating manufacturers. Orders approved by Global Fund on a rolling basis. ACTs distributed through normal distribution chain | Co-payments set by Global Fund and paid to manufacturer. 95% average subsidy level.  RRPs for adult pack USD 0.38-0.94 (no RRPs in Madagascar)  RRPs promoted in comms |  |
| Thomson 2014  Tanzania  AMFm v. no ACT subsidy | Pre-post without control  2010-2012  21 months | Urban and rural  Low to moderate | All private for-profit outlets within country could purchase subsidised ACTs  Some components of the study focus on 3 regions | See IE Team 2012, Tougher 2012 | See IE Team 2012, Tougher 2012 | See IE Team 2012, Tougher 2012 | - | See IE Team 2012, Tougher 2012 | See IE Team 2012, Tougher 2012 |  |
| 1.3 Interventions to enhance user adherence to subsidised ACTs | | | | | | | | | | |
| Bruxvoort 2014  Tanzania  Text message reminders to PMR staff v. no text message reminders | Cluster RCT  2012  2.5 months | Urban and rural  Moderate | Drug shops 40  Must have sold at least 5 ACTs in previous week, have a mobile phone, be accessible | - | - | - | Daily (M-F) text-messages to PMR staff for first 4 weeks, then 3 times/week for 10 weeks. Messages were reminders of advice for dispensing ACTs | - | (AMFm subsidised ACT available in both arms) |  |
| Cohen 2018  Uganda  Social marketing packaging v. stickers on manufacturer’s package v. manufacturer’s package | Individually randomised controlled trial  2010-2011  5 months | Rural  High | Drug shops 9  Must be licensed, open for >=1 yr, open long hours and most days, and have many customers and well-qualified staff | 1 day | - | - | Messages related to adherence on medicine packaging | ACTs brought to participating shops and dispensed by study team | Households provided with card allowing them to purchase ACTs for 2010 USD 0.09-0.35  25% of patients received RDTs as part of cross-cutting intervention (see Saran 2016) |  |
| Raifman 2014  Ghana  Long text message reminder v. short text message v. no text message | Individually randomised controlled trial  2011  5 months | Urban and rural  High | Public and private outlets distributing ACTs 84 (breakdown by type not reported)9  Must be within 30-min. drive from Tamale city-centre | - | - | - | Users messaged twice daily for 3 days to remind them to complete their full treatment course. | - | (AMFm subsidised ACT available in all arms) |  |
| 2. Introducing and enhancing RDT and ACT use | | | | | | | | | | |
| 2.1 RDTs conducted by PMRs | | | | | | | | | | |
| Ansah 2015  Ghana  RDTs v. no RDTs | Cluster RCT  2011-13  17 months | Rural  High | Drug shops 27  Must be licenced | 4 days (control arm received 3-day training which did not cover RDTs). New staff were trained in shop | Weekly visit for the 1st month, then another visit mid-trial | Community sensitization meetings | - | RDTs provided to outlets by study team  ACT provided through usual supply chain | RDTs provided free to drug shops and users  (AMFm subsidised ACT available in both arms) |  |
| Cohen 2015  Uganda  RDTs v. no RDTs | Cluster RCT  2011-12  9 months | Mostly rural  High | Drug shops 92  Must be registered | 2 days. New staff were invited to additional training | Monthly visit | - | - | RDTs purchased through designated wholesaler  ACT provided through usual supply chain | Subsidised RDT price to wholesaler USD 0.12; to drug shops USD 0.19 (first 40 provided to drug shops for free)  (AMFm subsidised ACT available in both arms) |  |
| Dieci 2023  Kenya  Patient subsidies v. Provider incentives v. Combined intervention v. No intervention | Cluster RCT  2021-22  8 months | Rural and urban  Low / low to moderate | Pharmacies 105  Must be licenced and active user of specific sales and inventory management  digital platform and manage their own stock of RDTs and treatments | Duration not stated | Outreach calls and site visits (frequency not stated) | - | - | RDTs and ACTs provided by programme in intervention arm. Control arm procures through usual channels. | Patient subsidy arm  RDT RRP USD 0.10  ACT RRP USD 0.29  Provider incentive arm:  Provider receives USD 0.86 for RDT, USD 0.76 for ACT following positive RDT, USD 0.29 for recording  Combined arm  RDT RRP USD 0.38  ACT RRP USD 0.57  Provider receives USD 0.14 for RDT, USD 0.14 for ACT following positive RDT, USD 0.29 for recording |  |
| Maloney 2017  Tanzania  Subsidised RDTs v. unsubsidised RDTs v. no RDTs | Pre-post with control  2013-14  12 months | Mostly rural  Moderate | Subsidised arm Drug shops 147  Unsubsidised arm  Drug shops 115  Must be Accredited Drug Dispensing Outlets (ADDOs) with licenced sellers | 2 days | Quarterly visit | - | - | RDTs purchased through designated wholesalers and importer  ACT provided through usual supply chain  Subsidised arm  ACT subsidy reimbursed to importer by study team | Subsidised arm  RDT RRP USD 0.32  Unsubsidised arm  RDT RRP USD 0.67  Fixed markups for wholesalers and importer  (CPM subsidised ACT available in all arms) |  |
| Mbonye 2015, Hutchinson 2017, Hansen 2017, antibiotic results reported in Hopkins 2017  Uganda  RDTs + ACT v. ACT only | Cluster RCT  2010-11  14 months | Urban and rural  Moderate | Drug shops 29  Must be registered | RDT & ACT arm  4 days  ACT only arm 3-day training | RDT & ACT arm  Weekly visits for 2 months, then scaled down to occasional support as requested | Both arms  Community  sensitization through meetings, roadside  signs and leaflets | - | RDT & ACT arm  ACT, rectal artesunate and RDTs provided by study team  ACT only arm  ACT & rectal artesunate provided by study team | ACT, rectal artesunate (both arms) and RDTs (intervention arm) provided free to drug shops.  RDT RRP USD 0.2  ACT RRP USD 0.4-1.2 |  |
| Omale 2021  Nigeria  Social group meetings + provider training v. Social group meetings v. No intervention | Cluster RCT  2018-19  3 months | Rural  High | Social group+ provider arm  Drug shops & Public clinics 42  Social group arm Drug shops & Public clinics 42  (no breakdown by provider type)  Must offer RDTs, and have had RDT training | Social group+ provider arm  1 day  Social group arm  No training | Social group+ provider arm  Monthly visit + min. of 2 mystery client visits  Social group arm  no supervision | Both intervention arms  Monthly discussions with social groups (local associations) | Both intervention arms  Weekly text messages to social group members  Social group+ provider arm  Twice-weekly text messages to providers | Both intervention arms  ACT and RDT provided through usual supply chain | -  (CPM terminated Q1 2018 so only limited quantities of subsidised ACT would have been available in both arms) |  |
| Onwujekwe 2015  Nigeria  RDT provision with: Provider+ school support v. Provider support v. Control (RDT demonstration) | Cluster RCT  2011  6 months | Urban and rural  Moderate | Provider+School arm  Drug shops, Pharmacies & Public clinics 74^4^ Provider arm  Drug shops, Pharmacies & Public clinics 110^4^  Control arm  Drug shops, Pharmacies & Public clinics 75^4^ | Provider+School and Provider arms  2 days  Control arm  RDT demonstration only | Both intervention arms  Monthly | Provider+School arm  Teachers trained to train student peer educators and conduct community awareness activities | - | Intervention and control arms  RDTs provided by study team  ACT provided through usual supply chain | Intervention and control arms  RDTs provided free to providers. Public facilities provided free to users  PMR RDT RRP USD 0.6  (AMFm subsidised ACT available in both arms) |  |
| Soniran 2022  Ghana  RDT subsidy and supporting interventions v. no intervention | Pre-post with control^11^  2019-20  12 months | Rural  Moderate | Drug shops 7 | 2 days | Quarterly | Community  Meetings led by CHWs and town criers | - | RDTs provided by study team  ACT provided through usual supply chain | RDTs provided free to drug shops  RDT RRP USD 0.44 |  |
| 2.2 RDTs conducted by study staff | | | | | | | | | | |
| Cohen 2015  Kenya  11 combinations of ACT and RDT subsidies of different levels v. no subsidy | Individually randomised controlled trial  2009  4 months | Rural  High/ Moderate | Drug shops 4  Must be far from health facilities and shops receiving other interventions, have many customers, and well-qualified staff | - |  |  | Direct communication to voucher recipients on effectiveness of ACTs, adherence to dosing regimen, and info on RDTs if provided a RDT voucher | Direct provision to outlets | 3 levels RDT subsidy:  100%: Free RDTs  85%: USD 0.19  85% but refundable conditional on positive test  3 levels of ACT subsidy (adult price):  92%: USD 0.50  88%: USD 0.75  80%: USD 1.25 |  |
| Ikwuobe 2013  Nigeria  RDTs v. no RDTs (users >=10 yrs) | Post intervention time series with control  2012  35 days | Urban  Moderate | Pharmacy 1  With sufficient daily anti-malarial sales to reach intended sample size | Duration not stated | - | - | - | RDTs provided and conducted by study team  ACT provided through usual supply chain | RDTs provided free to users  (AMFm subsidised ACT available in both arms) |  |
| Laktabai 2020  Kenya  2X2 factorial experiment comparing different levels of RDT subsidy and conditional ACT subsidy | Individually randomised controlled trial  2018-19  19 months | Rural  Low / low to moderate | Retail medicine outlets (similar to drug shops in other settings) 10  Must be registered and stock quality assured ACTs | - | - | - | - | RDTs provided and conducted by study team  ACT provided through usual supply chain  ACT retail subsidy reimbursed to PMRs by study team | RDT price to user:  Arm 1 and 2 50% subsidy to user (USD 0.2)  Arm 3 and 4  no subsidy (USD 0.4)  Conditional ACT subsidy:  Arm 1 and 3  Free to user  Arm 2 and 4  67% subsidy |  |
| Modrek 2014  Nigeria  Text messages to adults on which medicines to take post-RDT v. RDTs only | Individually randomised controlled trial  (Dates and duration not stated) | Urban and peri-urban  High/ moderate | Pharmacies, Drug shops (numbers not stated)  Excluded small drug retailers whose main business was not medicine sales | - | - | - | Text messages to RDT positive users reminding them to take ACT, and to RDT negative users reminding them not to take any antimalarials | RDTs provided and conducted by study team  ACT provided by study team to users testing positive | RDTs and ACTs provided for free to users |  |
| Saran 2016  Uganda  Free RDT + ACT subsidy v. ACT subsidy | Individually randomized controlled trial  2011  4 months | Rural  High | Drug shops 9  Must be licensed, open for >=1 yr, open long hours and most days, and have many customers and well-qualified staff | 1 day  (training only covered ACT; RDT conducted by study staff) | - | - | - | ACT & RDTs provided by study team; RDTs conducted by study team | Free RDT+ ACT subsidy arm  RDTs free to users  Both arms  95% ACT subsidy |  |
| 2.3 RDTs conducted by CHWs, with medicines provided by PMRs | | | | | | | | | | |
| O’Meara 2016  Kenya  2X2 factorial experiment comparing different levels of RDT subsidy and conditional ACT subsidy | Individually randomised controlled trial  2014-15  12 months | Rural  Low | Retail medicine outlets (similar to drug shops in other settings) 11  Must stock green leaf ACT | -  (3-day training for 20 CHWs conducting RDTs) | - | - | - | RDTs provided to CHWs by study team. CHW provided ACT voucher to patients testing positive redeemable at PMR  ACT provided through usual supply chain | RDT user price:  Arm A and B free  Arm C and D USD 0.5  Conditional ACT subsidy:  Arm A and C  USD 0.65 for adult  Arm B and D  USD 1.25 for adult (CPM subsidised ACTs available in all arms) |  |
| O’Meara 2018, Laktabai 2022  Kenya  Free RDT + conditional ACT subsidy v. no intervention | Cluster RCT  2015-18  20 months | Rural  Low / moderate | Retail medicine outlets (similar to drug shops in other settings) 42  Must stock stocked quality-assured ACT | -  (training for 292 CHWs conducting RDTs – no. of days not specified) | - | Meetings with community leaders, appearances at public meetings, printed posters in retail outlets | - | RDTs provided to CHWs by study team. CHW provided ACT voucher to patients testing positive redeemable at PMR  ACT provided through usual supply chain  ACT retail subsidy reimbursed to PMRs by study team | RDTs provided by CHWs for free to users  Conditional ACT subsidy redeemable at PMR of a third to a quarter of typical retail price  (CPM subsidised ACTs available in all arms) |  |
| 3. Introducing and enhancing iCCM | | | | | | | | | | |
| Awor 2014  Uganda  iCCM for U5s v. no intervention | Pre-post with control  2011-12  9 months | Rural  High | Drug shops 44  Must be registered | 5 days | Repeated visits (frequency not stated) | Communication  campaign through  shop branding,  public gatherings and radio | - | iCCM products^4^ (including RDTs and ACT) provided by study team | RDTs provided free to users.  Paediatric ACT provided to PMR for c. USD 0.25 to sell at USD 0.38  (AMFm subsidised ACT available from Q2-2011 in both arms) |  |
| Bagonza 2021  Uganda  iCCM with peer supervision for U5s v. standard iCCM for U5s | Interrupted time series with control  2016-7  8 months | Rural^3^  High | Drug shops 60  Must be registered | 3 day training for peer supervisors  (all drug shops in intervention and control areas had earlier received iCCM training) | Monthly visits by peer supervisors | - | - | All products provided by usual supply chain | -  (need to note whether CPM operational) |  |
| Kitutu 2017  Uganda  iCCM for U5s v. no intervention | Pre-post with control  2014-15  16 months | Rural  Low to moderate / moderate | Drug shops 61  Must be licenced and sell human rather than veterinary medicines | Yes (duration not stated) | Monthly | Community sensitisation through radio, workshops for CHWs, and posters in drug shops | - | iCCM products^5^ (including RDTs and ACT) provided by study team to designated wholesalers, who supplied drug shops | RDTs provided free to drug shops and users.  Subsidised ACT, provided to drug shops (subsidy level not stated)  (CPM subsidised ACT available in both arms) |  |
| Mbonye 2020  Uganda  iCCM for U5s v. no intervention | Cluster RCT  2015-17  26 months | Urban and rural  Moderate | Drug shops 35  Private clinics 10  Outlets must be registered and established for at least 12 months | 4 days  Additional quarterly meetings between public and private providers to discuss health system issues | Weekly for two months, then scaled down to occasional support as requested | Meetings with community leaders; leaflets; training Village Health Teams to mobilise communities | - | “timers, basic drugs, and supplies”  provided by study team (unclear if this included ACTs and RDTs) | Not stated  (CPM subsidised ACT available in both arms in 2015) |  |
| 4. Broader private sector strategies including ACT | | | | | | | | | | |
| Björkman Nyqvist 2019, Björkman Nyqvist 2021  Uganda  Villages with CHWs as retailers v villages without | Cluster RCT  Bjorkman 2021  2010-2011  18 months  Bjorkman 2019  2010-2013  3 years | Rural  High | CHWs as retailers  Bjorkman 2021  49  Bjorkman 2019  115  Women aged 18-45 with basic math and writing skills assigned to a village with <400 households | Initial training: 2 weeks  Refresher training: 1 day, monthly | - | - | - | CHWs purchase products from NGO branches.  152 products available to CHWs, including prevention goods, curative treatments (including ACTs), and other health-related commodities. | ACTs purchased by CHWs from the NGO at a wholesale price ~40% below the market price. RRPs set at 20-30% less the prevailing retail market price^10^  RRPs not stated. RRPs not printed on packs or promoted in comms  (AMFm subsidised ACT available from Q2-2011 in both arms) |  |
| Thomson 2018,  Tanzania  Drug shop accreditation v. no accreditation | Post with control  2003-2012  9 years | Urban and rural  Low to Moderate | Accreditation complete in 14/21 regions | Initial training: 35 days  Refresher: 1 day (2 regions only) | Yes, frequency not stated | - | - | - | (AMFm subsidised ACT available in all arms from Q4 2010) |  |
| Briggs 2014  Tanzania  Drug shop accreditation v. no accreditation | Post with control  2003-2012  9 years | Urban and rural  Low to moderate/ moderate | Accreditation complete in 15/21 regions, covering 3,800 shops | Initial training: 35 days  Refresher: 1 day (2 regions only) | Yes, frequency not stated | - | - | - | (AMFm subsidised ACT available in all arms from Q4 2010) |  |

^1^ Transmission is measured by the *P. falciparum* parasite rate among 2-10 year olds in the study areas at the time of the study (malariaatlas.org), classified as low <5%, low to moderate 5–9%, moderate 10–29% and high ≥30%

^2^ where intervention did not subsidise ACT directly we have noted whether the AMFm or Co-payment Mechanism was in operation in the country at that time

^3^ rural / urban status based on district name as not stated in paper

^4^ iCCM products generally comprised paediatric pre-packaged ACT, amoxicillin, ORS and zinc sulphate, RDTs, respiratory timers, and in some cases rectal artesunate

^5^ Subsidised ACTs were available from July 2009-Sept 2011 and then April 2012-March 2013

^6^ Details on product distribution provided by study authors

^7^ The study presents data prior to AMFm, but the focus on this paper is comparison of AMFm to the CPM. Therefore, dates and duration cover the CPM period. The data from this paper comparing pre-post AMFm are presented under Tougher 2012 only to avoid duplication

^8^ Communications activities were implemented at scale for more than 6 months in Ghana, Kenya, and Tanzania (Mainland and Zanzibar); less than 3 months in Madagascar, Niger and Nigeria; and not at scale in Uganda

^9^ Most patients in the study visited public and private facilities. 28% of patients came from drug shops

^10^ Description of markups comes from Björkman Nyqvist 2021, which involves data collection up to 2011. Pricing and markups may have differed at later stages of the intervention. However, specific details for ACTs are not provided in the study covering the longer period

^11^ Stated to be cluster randomised trial, but clusters are not randomly allocated to intervention arms
