# Supplementary material for: Improving malaria case management with artemisinin-based combination therapies and malaria rapid diagnostic tests in private medicine retail outlets in sub-Saharan Africa: A systematic review
Source: PLoS One. 2024 Jul 29;19(7):e0286718. doi: 10.1371/journal.pone.0286718 (PMC11285950; doi:10.1371/journal.pone.0286718)
Supplement: S4 Table — (DOCX) [file pone.0286718.s004.docx]

## S4 Table. Study data collection methods

## 4a: Interventions without diagnostics

| **Study** | **Outcomes** | | | | | | |
| --- | --- | --- | --- | --- | --- | --- | --- |
|  | **ACT uptake** | | |  |  |  |  |
|  | **ACT use among patients** | **ACT market share (use)** | **ACT market share (sales vols)** | **Antibiotic uptake** | **Patient adherence to treatment regimen** | **Medicine quality** | **Health outcomes** |
| **1. Introducing and enhancing ACT use (without diagnostics)** | | | | | | | |
| **1.1 Sub-national ACT subsidy programmes** | | | | | | | |
| Kangwana 2011, Kangwana 2013  Kenya | Household survey (Kangwana 2011) | Mystery shopper (Kangwana 2013) |  |  | Household survey  (Kangwana 2011) |  |  |
| Lussiana 2016  Angola |  |  | Outlet survey |  |  |  |  |
| Sabot 2009  Tanzania |  | Exit interview | Outlet survey |  |  |  |  |
| Talisuna 2012  Uganda | Exit interview | Exit interview |  |  | Exit interview |  |  |
| **1.2 National ACT subsidy programmes** | | | | | | | |
| ACTwatch 2017  Multiple |  |  | Outlet survey |  |  |  |  |
| Fink 2013  Uganda | Household survey | Household survey |  |  |  |  |  |
| Fiore 2018  Ghana, Nigeria, & Uganda | Household survey |  |  |  |  |  |  |
| IE Team 2012, Tougher 2012  Multiple | Household survey | Household survey | Outlet survey |  |  |  |  |
| Thomson 2014  Tanzania | Household survey | Household survey | Outlet survey |  |  |  |  |
| **1.3 Interventions to enhance user adherence to subsidised ACT** | | | | | | | |
| Bruxvoort 2014  Tanzania |  |  |  |  | Patient follow-up surveys |  |  |
| Cohen 2018  Uganda |  |  |  |  | Patient follow-up surveys |  |  |
| Raifman 2014  Ghana |  |  |  |  | Patient follow-up surveys |  |  |
| **4. Broader private sector strategies including ACT** | | | | | | | |
| Björkman Nyqvist 2019, Björkman Nyqvist 2021  Uganda | Household survey |  |  |  |  | Mystery shoppers (Bjorkman 2021) |  |
| Thomson 2018,  Tanzania |  |  | Outlet survey |  |  |  |  |
| Briggs 2014  Tanzania | Exit interviews |  |  | Exit interviews |  |  |  |

## 4b Study data collection methods (interventions with diagnostics)

| **Study** | **Outcomes** | | | | | |
| --- | --- | --- | --- | --- | --- | --- |
|  | **RDT uptake** | **ACT uptake** | **Antimalarial dispensing according to RDT result** | **Antibiotic uptake** | **Referral according to protocol** | **Patient adherence to treatment regimen** |
| **2. Introducing and enhancing RDT use** | | | | | | |
| **2.1 RDTs conducted by PMR** | | | | | | |
| Ansah 2015  Ghana | Provider records | Provider records | Provider records |  | Provider records | Patient follow-up |
| Cohen 2015  Uganda | Household survey | Household survey | Household survey | Household survey |  |  |
| Dieci 2023  Kenya | Digital sales records | Digital sales records | Digital sales records |  |  |  |
| Maloney 2017  Tanzania | Exit interviews | Exit interviews | Exit interviews | Exit interviews |  |  |
| Mbonye 2015, Hutchinson 2017, Hansen 2017  Uganda | Provider records | Provider records | Provider records | Patient follow-up survey |  |  |
| Omale 2021  Nigeria | Household survey |  |  |  |  |  |
| Onwujekwe 2015  Nigeria | Exit interviews | Exit interviews  and provider  records | Exit interviews  and provider  records | Exit  interviews  and provider  records |  |  |
| Soniran 2022  Ghana | Mystery shoppers^2^ | Mystery shoppers | Mystery shoppers |  |  |  |
| **2.2 RDTs conducted by study team** | | | | | | |
| Cohen 2015  Kenya | Household survey | Household survey | Study staff records | Household survey |  |  |
| Ikwuobe 2013  Nigeria | Study staff records | Study staff records | Study staff records |  |  |  |
| Laktabai 2020  Kenya | Study staff records | Exit interviews | Exit interviews |  |  |  |
| Modrek 2014  Nigeria |  |  | Patient follow-up survey (by phone) |  |  |  |
| Saran 2016  Uganda |  |  |  |  |  | Patient follow-up |
| **2.3 RDTs conducted by CHWs, with medicines provided by PMRs** | | | | | | |
| O’Meara 2016  Kenya | CHW records | Patient follow- up survey | Patient follow- up survey |  |  |  |
| O’Meara 2018, Laktabai 2022  Kenya | Household survey | Household survey | Household survey | Household survey |  |  |
| **3. Introducing and enhancing iCCM** | | | | | | |
| Awor 2014  Uganda | Exit interviews^1^ | Exit interviews^1^ | Observation | Exit interviews |  |  |
| Bagonza 2021  Uganda |  |  | Provider records |  |  |  |
| Kitutu 2017  Uganda | Exit interviews | Exit interviews |  |  |  |  |
| Mbonye 2020  Uganda | Provider records | Provider records | Provider records |  |  |  |

^1^ We present data from exit interviews; this indicator was also collected through household survey and direct observation

^2^ Soniran also have household survey data on RDT uptake but these are only available for the intervention arm so we present the mystery shopper data
