# Supplementary material for: Improving malaria case management with artemisinin-based combination therapies and malaria rapid diagnostic tests in private medicine retail outlets in sub-Saharan Africa: A systematic review
Source: PLoS One. 2024 Jul 29;19(7):e0286718. doi: 10.1371/journal.pone.0286718 (PMC11285950; doi:10.1371/journal.pone.0286718)
Supplement: S5 Table — (DOCX) [file pone.0286718.s005.docx]

## S5 Table. Quality assessment of included studies

|  | **Reporting** | | | **External validity** | | **Internal validity** | | | | | | |
| --- | --- | --- | --- | --- | --- | --- | --- | --- | --- | --- | --- | --- |
| **First author, year published** | **1) Are the characteristics of the PMR included in the study clearly described?** | **2) Are the interventions of interest clearly described?** | **3) Have the characteristics of PMR lost to follow-up been described?** | **4) Were those subjects who participated representative of the target population?** | **5) Does the implementation of the intervention reflect what would happen in regular practice?** | **6) Were the statistical tests used to assess the main outcomes appropriate?** | **7) Was compliance with the intervention/s reliable?** | **8) Was the length of follow-up adequate?** | **9) Were there no important differences between groups prior to the intervention?** | **10) Were study subjects randomised to intervention groups?** | **11) Was there adequate adjustment for confounding in the analyses from which the main findings were drawn?** | **12) Did the study have sufficient power to detect a clinically important effect?** |
| **1. Introducing and enhancing ACT use (without diagnostics)** | | | | | | | | | | | | |
| **1.1 Sub-national ACT subsidy programmes** | | | | | | | | | | | | |
| Kangwana 2011 | Yes | Yes | Yes | Yes | Yes | Yes | Yes | Yes | Yes | Yes | Yes | Yes |
| Kangwana 2013 | Yes | Yes | Yes | Yes | Yes | Yes | Yes | Yes | Yes | Yes | Yes | Yes |
| Lussiana 2016 | Yes | Yes | Yes | No | Yes | No | Yes | Yes | n/a | No | No | No |
|  |  |  |  | Analysis restricted to an unrepresentative subset of PMR |  | No statistical testing done for outcomes that were extracted |  |  | Before & after study with no control | Not randomised | No adjustment done | No sample size calculation reported |
| Sabot 2009 | Yes | Yes | Yes | Yes | Yes | Yes | Yes | Yes | Yes | No | Yes | No |
|  |  |  |  |  |  |  |  |  |  | One cluster randomly allocated to each arm |  | No sample size calculation reported |
| Talisuna 2012 | Yes | Yes | Yes | Yes | Yes | Yes | Yes | Yes | Yes | No | Yes | No |
|  |  |  |  |  |  |  |  |  |  | Not randomised |  | No sample size calculation reported |
| **1.2 National ACT subsidy programmes interventions** | | | | | | | | | | | | |
| ACTwatch 2017 | Yes | No | N/A | Yes | Yes | Yes | Yes | Yes | n/a | No | Yes | Yes |
|  |  | Certain details of intervention components not explained | Repeated cross-section |  |  |  |  |  | Before & after study with no control | Not randomised |  |  |
| Fink 2013 | Yes | No | N/A | No | Yes | Yes | Yes | Yes | n/a | No | Yes | No |
|  |  | Details of intervention components not described | Repeated cross-section | Only villages with registered drug shops included |  |  |  |  | Before & after study with no control | Not randomised |  | No sample size calculation reported |
| Fiore 2018 | Yes | No | N/A | Yes | Yes | Yes | No | Yes | Yes | No | Yes | No |
|  |  | Details of intervention components not described | Repeated cross-section |  |  |  | AMFm products present in some non-AMFm countries |  |  | Not randomised |  | No sample size calculation reported |
| IE Team 2012; Tougher 2012 | Yes | Yes | N/A | Yes | Yes | Yes | Yes | Yes | n/a | No | Yes | Yes |
|  |  |  | Repeated cross-section |  |  |  |  |  | Before & after study with no control | Not randomised |  |  |
| Thomson 2014 | Yes | Yes | N/A | Yes | Yes | Yes | Yes | Yes | n/a | No | Yes | Yes |
|  |  |  | Repeated cross-section |  |  |  |  |  | Before & after study with no control | Not randomised |  |  |
| **1.3 Interventions to enhance user adherence to subsidised ACT** | | | | | | | | | | | | |
| Bruxvoort 2014 | Yes | Yes | Yes | Yes | Yes | Yes | Yes | No | Yes | Yes | Yes | Yes |
|  |  |  |  |  | Blister packs provided for free to dispenser, but patients paid the rec. prices |  |  | Total duration of the study is 14 weeks |  |  |  |  |
| Cohen 2018 | Yes | Yes | Yes | Yes | No | Yes | Yes | Yes | Yes | Yes | Yes | Yes |
|  |  |  |  |  | Subsidized ACT brought by survey team, free RDTs administered by survey team, and voucher delivered to households |  |  |  |  |  |  |  |
| Raifman 2014 | Yes | Yes | Yes | No | Yes | Yes | Yes | Yes | Yes | Yes | Yes | Yes |
|  |  |  |  | Excluded patients more likely to be in lower quintiles and have household heads with lower educational attainment |  |  |  |  |  |  |  |  |
| **2. Introducing and enhancing RDT and ACT use** | | | | | | | | | | | | |
| **2.1 RDTs conducted by PMRs** | | | | | | | | | | | | |
| Ansah 2015 | Yes | Yes | Yes | Yes | No | Yes | Yes | Yes | Yes | Yes | Yes | Yes |
|  |  |  |  |  | RDTs were supplied for free |  |  |  |  |  |  |  |
| Cohen 2015 | Yes | Yes | Yes | Yes | Yes | Yes | No | Yes | Yes | Yes | Yes | Yes |
|  |  |  |  |  |  |  | Not reported |  |  |  |  |  |
| Dieci 2023 | Yes | No | Yes | Yes | No | Yes | Yes | Yes | Yes | Yes | Yes | Yes |
|  |  | Duration of training not stated |  | Though must be active user of specific sales and inventory management  digital platform | RDTs and ACTs provided to intervention pharmacies by project |  |  |  |  |  |  |  |
| Maloney 2017 | Yes | Yes | Yes | Yes | Yes | Yes | Yes | Yes | No | Yes | Yes | Yes |
|  |  |  |  |  |  |  |  |  | at baseline parasitic diagnosis before attending PMR was 19% in intervention v. 3% in control, but no RDT use at PMR at baseline in either group |  |  |  |
| Mbonye 2015 | Yes | Yes | Yes | Yes | No | Yes | Yes | Yes | Yes | Yes | Yes | Yes |
|  |  |  |  |  | RDTs and ACTs were provided for free to drug shop owners |  |  |  |  |  |  |  |
| Hutchinson 2017 | Yes | Yes | Yes | Yes | No | No | Yes | Yes | Yes | Yes | No | No |
|  |  |  |  |  | RDTs and ACTs were provided for free to drug shop owners | No statistical tests presented |  |  |  |  |  | No sample size calculation reported |
| Hansen 2017 | Yes | Yes | No | Yes | No | Yes | No | Yes | Yes | Yes | Yes | Yes |
|  |  |  | No loss to follow up was described |  | RDTs and ACTs were provided for free to drug shop owners |  | Not reported |  |  |  |  |  |
| Omale 2021 | Yes | Yes | Yes | Yes | Yes | Yes | Yes | Yes | Yes | Yes | Yes | Yes |
| Onwujekwe 2015 | Yes | Yes | Yes | Yes | Yes | Yes | No | Yes | Yes | Yes | Yes | Yes |
|  |  |  |  |  |  |  | Unable to determine |  |  |  |  |  |
| Soniran 2022 | No | Yes | Yes | No | No | Yes | Yes | Yes | No | No | No | No |
|  | Inclusion criteria not described apart from retailer type |  |  | Unable to determine as not clear how OTCMS selected | RDTs provided to OTCMS by project for free |  |  |  | Some differences in baseline characteristics | Intervention clusters are all in one district | No adjustment done | No sample size calculation reported; very low sample size for mystery shoppers |
| **2.2 RDTs conducted by study staff** | | | | | | | | | | | | |
| Cohen 2015 | Yes | Yes | Yes | Yes | No | Yes | Yes | No | Yes | Yes | Yes | Yes |
|  |  |  |  |  | Direct door-to-door distribution of vouchers to households; study team embedded in PMR |  |  | 4 months |  |  |  |  |
| Ikwuobe 2013 | Yes | No | Yes | Yes | No | Yes | Yes | No | Yes | Yes | Yes | Yes |
|  |  | Role of study nurse not described clearly |  |  | Free RDTs were provided; testing conducted by study nurse |  |  | 2-3 months |  |  |  |  |
| Laktabai 2020 | Yes | Yes | Yes | Yes | No | Yes | Yes | Yes | Yes | Yes | Yes | Yes |
|  |  |  |  |  | RDTs supplied by study team |  |  |  |  |  |  |  |
| Modrek 2014 | Yes | Yes | Yes | No | No | Yes | No | No | Yes | Yes | Yes | No |
|  |  |  |  |  | RDTs conducted by study nurse; ACTs and RDTs provided free to patients |  | No stated |  |  |  |  | No sample size calculation reported |
| Saran 2016 | Yes | Yes | Yes | Yes | No | Yes | Yes | No | Yes | Yes | Yes | Yes |
|  |  |  |  |  | RDT supplied for free; vouchers delivered directly to households; RDTs performed by study team |  |  | 4 months |  |  |  |  |
| **2.3 RDTs conducted by CHWs, with medicines provided by PMRs** | | | | | | | | | | | | |
| O’Meara 2016 | Yes | Yes | Yes | No | No | Yes | Yes | Yes | Yes | Yes | Yes | Yes |
|  |  |  |  | Enrolled could differ systematically from overall febrile population | Direct door-to-door distribution of vouchers to households |  |  |  |  |  |  |  |
| O’Meara 2018, Laktabai 2022 | Yes | No | Yes | Yes | Yes | Yes | Yes | Yes | Yes | Yes | Yes | Yes |
|  |  | Duration of training not reported |  |  |  |  |  |  |  |  |  |  |
| **3. Introducing and enhancing iCCM** | | | | | | | | | | | | |
| Awor 2014 | Yes | No | No | Yes | No | Yes | Yes | Yes | Yes | No | Yes | Yes |
|  |  | Product distribution mechanism not stated | Not stated whether any PMR lost to follow-up |  | RDT were provided for free |  |  |  |  | Not randomised |  |  |
| Bagonza 2021 | Yes | Yes | No | Yes | Yes | Yes | Yes | Yes | Yes | No | N/A | No |
|  |  |  | Not stated whether any PMR lost to follow-up |  |  |  |  |  |  | One cluster randomly allocated to each arm |  | No sample size calculation reported |
| Kitutu 2017 | Yes | Yes | No | Yes | No | Yes | Yes | Yes | No | No | Yes | Yes |
|  |  |  | Not stated whether any PMR lost to follow-up |  | RDTs provided for free |  |  |  | Some differences between arms at baseline though generally well-balanced | Not randomised |  |  |
| Mbonye 2020 | Yes | Yes | Yes | Yes | No | Yes | Yes | Yes | Yes | Yes | Yes | Yes |
|  |  |  |  |  | ACT and RDTs provided for free |  |  |  |  |  |  |  |
| **4. Broader private sector strategies including ACT** | | | | | | | | | | | | |
| Bjorkman Nyqvist 2019 | Yes | Yes | No | No | Yes | Yes | Yes | Yes | Yes | Yes | Yes | Yes |
|  |  |  | Unclear whether there were any PMR lost to follow-up | Study only includes villages with <400 households |  |  |  |  |  |  |  |  |
| Björkman Nyqvist 2021 | Yes | No | Yes | Yes | Yes | Yes | Yes | Yes | Yes | Yes | Yes | Yes |
|  | Cites detailed information in 2019 paper | Certain aspects of the intervention not reported (e.g. training) |  |  |  |  |  |  |  |  |  |  |
| Thomson 2018 | Yes | Yes | N/A | Yes | Yes | No | Yes | Yes | Yes | No | Yes | No |
|  |  |  | Repeated cross-section |  |  | Methods for analysis of market share outcome not reported |  |  |  | Not randomised |  | No sample size calculation reported |
| Briggs 2014 | Yes | Yes | N/A | Yes | Yes | Yes | Yes | Yes | No | No | Yes | Yes |
|  |  |  | Repeated cross-section |  |  |  |  |  | Only 1 intervention region and 1 control; could be unobserved differences between regions |  |  |  |
