# Supplementary material for: Improving malaria case management with artemisinin-based combination therapies and malaria rapid diagnostic tests in private medicine retail outlets in sub-Saharan Africa: A systematic review
Source: PLoS One. 2024 Jul 29;19(7):e0286718. doi: 10.1371/journal.pone.0286718 (PMC11285950; doi:10.1371/journal.pone.0286718)
Supplement: S6 Table — (DOCX) [file pone.0286718.s006.docx]

S6 Table. Heat maps

Table 6a and b present Heat maps summarising the interventions and main results for the studies without RDTs (6a) and with RDTs (6b). Note that the results in Table 6a are presented in terms of differences between intervention and contemporaneous or historical control. By contrast, results in Table 6b are presented in terms of levels in the intervention arms, because many of the key variables such as dispensing by test result, cannot be presented for baseline /control where very few RDTs were used.

## 6a: Heat map for Introducing and enhancing ACT use (without diagnostics) and Broader private sector strategies including ACT

| **Intervention intensity** | | **Change in intervention arm(s)** | |
| --- | --- | --- | --- |
|  | High |  | Significant improvement |
|  | Moderate |  | Large improvement, no significance testing |
|  | Low |  | Non-significant improvement (over 15 percentage pts) |
|  | Component not included |  | Non-significant improvement |
|  | Component included but intensity unknown |  | No improvement |

| Study Design | | Intervention components | | | | | Change in intermediate outcome | | | Change in primary outcomes ^1^ | | | |
| --- | --- | --- | --- | --- | --- | --- | --- | --- | --- | --- | --- | --- | --- |
| First author,  Yr published;  Country; | Comparison | Training | Supervision | Comms | Direct  product distribution to outlets | RRP for ACT (adult)^17^ | Price | Availability | ACT uptake | | | | Adherence |
|  |  |  |  |  |  |  | Change in ACT price | % pt change in availability of ACT | % pt change in febrile patients receiving  ACT | | % pt change in febrile patients receiving an antimalarial that received an ACT | % pt change in antimalarials dispensed that were ACTs | % pt change in patients dispensed ACT who complete the full dose as directed |
| 1. Introducing and enhancing ACT use (without diagnostics) | | | | | | | | | | | | | |
| 1.1 Sub-national ACT subsidy programmes | | | | | | | | | | | | | |
| Kangwana 2011, 2013  Kenya | Subsidised paediatric ACTs v. no subsidy | 1 day | Quarterly | Yes, mass | Yes, to outlets | $0.24 |  | 31.7*  (22.0-41.3) | 26.4* ^2^  (12.6-40.2) | | 23.6*^2 3^  (18.7 -28.6) |  | 17.6 ^2^  (-0.9 - 36.1) |
| Lussiana 2016  Angola | Subsidised paediatric ACTs v. before subsidy | Duration not stated | Monthly | Yes, mass | Yes, to outlets | $1.73 |  |  |  | |  | 52.8~ ^4^  (CI not stated) |  |
| Sabot 2009  Tanzania | Subsidised ACT with RRPs v. no subsidy | 1 day |  | Yes, mass | Yes, to wholesalers | $1.21 |  |  |  | | 64.2~ ^5 6^  (CI not stated) | Control not presented |  |
|  | Subsidised ACTs with RRP v. no subsidy | 1 day |  | Yes, mass | Yes, to wholesalers |  |  |  |  | | 34.7~ ^5 6^  (CI not stated) |  |  |
| Talisuna 2012  Uganda | Subsidised ACTs v. no subsidy | Duration not |  | Yes, mass | Yes, to outlets | $0.45 |  |  | 20.6* ^5^ (CI not stated) | | Control not presented |  | Control not presented |
| 1.2 National ACT subsidy programmes interventions | | | | | | | | | | | | | |
| ACTwatch 2017; IE Team 2012; Tougher 2012  Ghana | AMFm v. before AMFm | Duration ns | Duration ns | Yes, mass |  | $1.12 | -$2.30* | 57.8* ^7^  (51.7-63.8) |  | |  | 45.3* ^7^  (40.3-50.4) |  |
| Kenya | CPM 2014 v. before AMFm | Duration ns |  | Yes, mass |  | RRP ns | -$1.20 ^5 7^ | 50.0 ^5 7^ (CI not stated) |  | |  | 36.1~ ^5 7^  (CI not stated) |  |
|  | AMm v. before AMFm | Duration ns | Duration ns | Yes, mass |  | $0.55 | -$2.05* | 38.9* ^7^  (29.7-48.2) |  | |  | 49.3* ^7^  (39.5-59.1) |  |
| Madagascar | CPM 2015 v. before AMFm | Duration ns |  | Yes, mass |  | RRP ns | +$0.90 ^5 7^ | 3.2 ^5 7^ (CI not stated) |  | |  | 0.2~ ^5 7^  (CI not stated) |  |
|  | AMFm v. before AMFm | Duration ns |  | Yes, mass |  |  | +$0.46* ^7^ | 1.0 ^7^  (-3.3-5.4) | 5.0* ^8^  (0.3-9.7) | | 37.3~ ^58^  (CI not stated) | 15.1* ^7^  (5.6-24.6) |  |
| Niger | AMFm v. before AMFm |  | Duration ns | Yes, mass |  | $0.82 | -$1.28* ^7^ | 7.6 * ^7^  (5.6-10.6) |  | |  | 14.4* ^7^  (8.4-20.0) |  |
| Nigeria | CPM 2015 v. before AMFm | Duration ns |  | Yes, mass |  | RRP ns | -$2.70 ^5 7^ | 57.3 ^5 7^  (CI not stated) |  | |  | 32.8~ ^5 7^  (CI not stated) |  |
|  | AMFm v. before AMFm |  |  | Yes, mass |  | $0.70 | -$2.99* ^7^ | 26.3* ^7^  (15-1-37.5) | 6.7* ^8^  (4.2-9.2) | | 16.4~ ^5 8^  (CI not stated) | 15.6* ^7^  (12.1-19.1) |  |
| Tanzania - Mainland | CPM v. before AMFm | Duration ns |  | Yes, mass |  | RRP ns | -$4.00 ^5 7^ | 68.0 ^5 7^  (CI not stated) |  | |  | 37.0~ ^5 7^  (CI not stated) |  |
|  | AMFm v. before AMFm | Duration ns |  | Yes, mass |  | $1.36 | -$4.34* ^7^ | 55.6* ^7^  (46.4-64.8) | -4.2~ ^5 8^  (CI not stated) | | -1.6~ ^5 8^  (CI not stated) | 30.0* ^7^  (21.9- 38.1) |  |
| Tanzania – Zanzibar | AMFm v. before AMFm | Duration ns |  | Yes, mass |  | $0.83 | -$4.82  (No CI) | 71.3* ^7^  (No CI) |  | |  | 58.7~ ^7^  (No CI) |  |
| Uganda | CPM v. before AMFm | Duration ns |  |  |  | RRP ns | -$1.25 ^5 7^  (CI not stated) | 71.0 ^5 7^ (CI not stated) |  | |  | 42.4~ ^5 7^  (CI not stated) |  |
|  | AMFm v. before AMFm |  |  |  |  | $0.47 | -$0.83 | 54.2* ^7^  (47.3-61.0) | 24.0* ^8^  (15.5-32.5) | | 42.5~ ^5 8^  (CI not stated) | 33.4* ^7^  (26.0-40.8) |  |
| Fink 2013  Uganda | AMFm v. before AMFm |  |  |  |  | $0.47 | -511 Ush*^8 16^  (CI not stated) |  | 10.3* ^8^  (CI not stated) | | 14.5 * (CI not stated) |  |  |
| Fiore 2018 ^16^  Pooled | CPM/AMFm v. no subsidies | Duration ns |  | Yes, mass |  | RRP ns |  |  | 6.8 * **^9^**  (3.4) | |  |  |  |
| Fiore 2018 ^16^  Ghana | CPM/AMFm v. no subsidies | Duration ns |  | Yes, mass |  | RRP ns |  |  | 0.4 **^9^**  (3.8) | |  |  |  |
| Fiore 2018 ^16^  Nigeria | CPM/AMFm v. no subsidies | Duration ns |  | Yes, mass |  | RRP ns |  |  | 2.1 **^9^**  (1.9) | |  |  |  |
| Fiore 2018 ^16^  Uganda | CPM/AMFm v. no subsidies | Duration ns |  |  |  | RRP ns |  |  | 19.1* **^9^** (4.6) | |  |  |  |
| Thomson 2014  Tanzania | AMFm v. before AMFm | Duration ns |  | Yes, mass |  | $1.36 | -$4.69* ^5 11^  (CI not stated) | 56.8* ^5 11^  (CI not stated_ | 8.4* ^5 10^  (CI not stated) | | 22.1* ^5 10^  (CI not stated) | 31.8 * ^11^  (CI not stated) |  |
| 1.3 Interventions to enhance user adherence to subsidised ACT | | | | | | | | | | | | | |
| Bruxvoort 2014  Tanzania | Text message reminders to PMR staff v. no reminder |  |  | Yes, individ. |  |  |  |  |  | |  |  | 1-1.5 ^5^  (CI not stated) |
| Cohen 2018  Uganda | Social marketing packaging v. manufacturer’s package | 1 day |  | Yes,  individ.  (on pack) | Yes, to outlets |  |  |  |  | |  |  | -2.7  [2.6] |
|  | Stickers on manufacturer’s package v. manufacturer’s package | 1 day |  | Yes,  individ (on pack) | Yes, to outlets |  |  |  |  | |  |  | 5.7 ^12^  [3.0] |
| Raifman 2014  Ghana | Long text message v. no reminder |  |  | Yes, individ |  |  |  |  |  | |  |  | 4.3 ^5 13^  (CI not stated) |
|  | Any text message v. no reminder |  |  | Yes, individ |  |  |  |  |  | |  |  | -0.9 ^5 13^  (CI not stated) |
| 4. Broader private sector strategies including ACT | | | | | | | | | | | | | |
| Björkman Nyqvist 2019 & 2021  Uganda | Villages with CHWs as retailers v. villages without (2021) | 2 weeks |  |  |  | RRP ns | -1,510 ^5^ Ush* | -7.7  [7.1] | -2.4 ^8^  [6.8] | |  |  |  |
|  | Villages with CHWs as retailers v. villages without (2019) | 2 weeks |  |  |  | RRP ns |  |  | 0.4~ ^8^  [1.5] | |  |  |  |
| Thomson 2018,  Tanzania | Drug shop accreditation v. no accreditation | 35 days | Yes |  |  |  | -$0.11* ^5 14^ | 3.0 ^5 14^  (CI not stated) |  | |  | 16.5 ^5 14^  (CI not stated) |  |
| Briggs 2014  Tanzania | Drug shop accreditation v. no accreditation | 35 days | Yes |  |  |  |  |  | 8.0~ ^5 15^  (CI not stated) | |  |  |  |

* p<0.05 for significance of difference from control or baseline; ~ significance not reported. Confidence intervals shown in () if reported, [] shows standard errors if reported instead of confidence intervals.

^1^ No studies were identified reporting on outcomes related to referral on the basis of protocol. 1 study reported on each of antibiotic uptake, antimalarial quality, and health outcomes.^2^ Outcomes is measured among febrile **children 3-59 months** using **AL**.

^3^ % of **mystery shoppers** dispensed **AL**.

^4^ Proportion of volumes sold that were **subsidised AL.** Comparison is from period without subsidised ACTs (2012) to a period with subsidised ACTs (2013). Results for other time points are presented, and are broadly similar.

^5^ Authors’ calculation.

^6^ Outcome is measured among febrile **children <5**. Results from febrile adults show higher use of ACTs in the intervention arm without an RRP compared to the intervention arm with an RRP.

^7^ Outcome is price, availability or market share of **quality-assured ACTs** among all **private for-profit outlets**, including private facilities.

^8^ Outcome is measured among febrile **children <5**

^9^ Outcome is febrile **children** **< 5** that received an ACT **from any private outlet**.

^10^ Outcome is measured among all febrile patient that sought care at a **specialised drug seller**.

^11^ Outcome is price, availability, or market share of **quality-assured ACTs** among **specialised drug sellers**.

^12^ Results are pooled for two versions of the sticker message. One sticker message “Malaria is not gone until…” had a statistically significant effect on adherence compared to the manufacturer’s standard packaging.

^13^ Measured among drug shop clients.

^14^ Outcome is price, availability, or market share of **AMFm-subsidised** **AL** in **drug shops.**

^15^ Outcome is measured among **drug shop** **clients**

^16^ Intervention components were not reported by Fiore 2018. Intervention components filled in as present if reported in either the AMFm or CPM period in Ghana, Nigeria, or Uganda in Tougher 2012 or ACTwatch 2017.

^17^ RRPs have been adjusted to 2021 USD from the original USD amount as reported in the original paper using the US annual CPI from the World Bank. If the intervention only subsidised paediatric formulations, the RRP for the largest package size was adjusted to the price per adult-equivalent treatment dose (AETD).

## 6b: Heat Map for Introducing and enhancing RDT and ACT use; and Introducing and enhancing iCCM

| **Intervention intensity** | | **Levels in intervention arm(s)** | | **Data collection method** | |
| --- | --- | --- | --- | --- | --- |
|  | High |  | >=75% | Provide records or observation (likely high Hawthorne effect) |  |
|  | Moderate |  | >=50% & <75% |  |  |
|  | Low |  | >=25% & <50% | Other | (no shading) |
|  | Component not included |  | <25% |  |  |

| **Study Design** | | | **Intervention** | | | | | | | | | **Levels in intervention arms** | | | |
| --- | --- | --- | --- | --- | --- | --- | --- | --- | --- | --- | --- | --- | --- | --- | --- |
| First author,  Country | | Comparison | PMR training | PMR supervision | Mass comms | Individual comms | Direct distribution of ACT | Direct distribution of RDT | ACT subsidy level | | RDT subsidy level | RDT uptake | Antimalarial dispensing according to RDT result | | |
|  | |  |  |  |  |  |  |  |  | |  | % febrile patients receiving  RDT | % negative  not  receiving  antimalarial | %  positive  receiving ACT | % treated according to test result |
| **2. Introducing and enhancing RDT and ACT use** | | | | | | | | | | | | | | | |
| **2.1 RDTs conducted by PMRs** | | | | | | | | | | | | | | | |
| Ansah 2015 Ghana | | Trained providers and free RDTs | 4 days | Weekly for one month | YES |  |  | YES |  | | Free | 100  (provided to all) | 97 | 92 |  |
| Cohen 2015  Uganda | | Trained providers  & sub’d RDTs | 2 days | Monthly |  |  |  | Through designated wholesaler |  | | Sub | 17.5 | 56.3 | 40.9 |  |
| Dieci 2023  Kenya | Patient subsidies | | Duration not stated | Frequency not stated |  |  | YES | YES | | Subsidy of 80% | Subsidy of 90% | 35.3 | 90.2 | 90.8 |  |
|  | Provider incentives | | Duration not stated | Frequency not stated |  |  | YES | YES | |  |  | 27.7 | 89.3 | 85.7 |  |
|  | Combined intervention | | Duration not stated | Frequency not stated |  |  | YES | YES | | Subsidy of 60% | Subsidy of 60% | 27.9 | 82.2 | 87.4 |  |
| Maloney 2017  Tanzania | | Trained providers and sub’d RDTs | 2 days | Quarterly |  |  |  | Through designated wholesalers & importer |  | | 50% sub | 67  (CI 58–71) | 90.8^2^ | 84.4^2^ | not stated |
|  | | Trained providers and unsub’d RDTs | 2 days | Quarterly |  |  |  | Through designated wholesalers & importer |  | |  | 66  (CI 59–72) | 95.0^2^ | 67.3^2^ | not stated |
| Mbonye 2015  Uganda | | Trained providers and sub’d RDTs | 4 days v 3 days in control | Weekly for 2 months (both arms) | YES (both arms) |  | YES | YES | free to PMR, sub’d for user | | free to PMR, sub to user | 97.7 | 98.54 | 99.04 | 98.84 |
| Omale 2021  Nigeria | | Social group meetings, provider training | 1 day | Monthly | YES | YES |  |  |  | |  | 64.1 |  |  |  |
|  | | Social group meetings |  |  | YES | YES |  |  |  | |  | 62.9 |  |  |  |
| Onwujekwe 2015  Nigeria | | Trained providers, sub’d RDTs, school intervention | 2 days | Monthly | YES |  |  | YES |  | | free to PMR, sub to user | 8.4 | 13**^2^** | 71**^2^** | not stated |
|  | | Trained providers, sub’d RDTs | 2 days | Monthly | YES |  |  | YES |  | | free to PMR, sub to user | 12.3 | 43**^2^** | 80**^2^** | not stated |
| Soniran 2022  Ghana | Trained providers, sub’d RDTs | | 2 days | Quarterly | YES |  |  | YES | |  | free to PMR, sub to user | 38.1  (NB low n=42) | **83.3**  (NB low n=12) | 75  (NB low n=4) | 81.3  (NB low n=16) |
| **2.3 RDTs conducted by study staff** | | | | | | | | | | | | | | | |
| Cohen 2015  Kenya | | Any ACT subsidy^9^ |  |  |  | YES | YES | YES | Subsidy of 92%, 88% or 80% | | Free or 85% sub or no sub | 20.0 | 30 (92% sub)  40 (88% sub)  45 (80% sub)^1^ | 98 (92% sub)  98 (88% sub)  98 (80% sub)^1^ | not stated |
|  | | Any RDT subsidy^9^ |  |  |  | YES | YES | YES | Subsidy of 92%, 88% or 80% or no sub | | Free or 85% sub | 29.1 | not stated | not stated | not stated |
| Ikwuobe 2013  Nigeria | | Free RDTs | Duration not stated |  |  |  |  | Provided & conducted by study team |  | | Free | 100 (provided to all) | 48.4 | 85.7 | not stated |
| Laktabai 2020  Kenya | | 50% RDT subsidy and 100% ACT subsidy |  |  |  |  |  | Provided & conducted by study team | Free | | 50% sub | 98.6 | 94.9^4^ | 77.4 | 92.2^4^ |
|  | | 50% RDT subsidy and 67% ACT subsidy |  |  |  |  |  | Provided & conducted by study team | 67% sub | | 50% sub | 100 | 93.9^4^ | 87.0 | 92.4^4^ |
|  | | No RDT subsidy and 100% ACT subsidy |  |  |  |  |  | Provided & conducted by study team | Free | |  | 96.2 | 93.5^4^ | 92.2 | 93.2^4^ |
|  | | No RDT subsidy and 67% ACT subsidy |  |  |  |  |  | Provided & conducted by study team | 67% sub | |  | 96.5 | 91.0^4^ | 84.8 | 89.5^4^ |
| Modrek 2014  Nigeria | | Text messages to adult patients, free RDT and ACT |  |  |  | YES | Provided & conducted by study team | Provided & conducted by study team | Free | | Free | 100  (provided to all) | 76.9^5^ |  | 79.8^5^ |
| Saran 2016  Uganda | | Free RDT and ACT subsidy | 1 day |  |  |  | YES | Provided & conducted by study team | 95% sub | | Free |  |  |  |  |
| **2.3 RDTs conducted by CHWs, with medicines provided by PMRs** | | | | | | | | | | | | | | | |
| O’Meara 2016  Kenya | | Free RDT and 50% ACT subsidy | 3 day for CHW but not PMR |  |  |  |  | Provided to CHW | 50% sub | | Free | 71.1 | 72.5^4^ | 81.8 | 76.2^4^ |
|  | | Free RDT and no ACT subsidy | 3 day for CHW but not PMR |  |  |  |  | Provided to CHW |  | | Free | 67.0 | 80.0^4^ | 71.4 | 77.6^4^ |
|  | | No RDT subsidy and 50% ACT subsidy | 3 day for CHW but not PMR |  |  |  |  | Provided to CHW | 50% sub | |  | 42.5 | 87.1^4, 9^ | 84.0^10^ | 85.7^4^ |
| O’Meara 2018  Kenya | | Free RDT and ACT subsidy | for CHW but not for PMR |  | YES |  |  | Provided to CHW | Sub 66-75% | | Free | 55.0^3^ | 70.1^4^ | 90.0 | 88.5^4^ |
| **3. Introducing and enhancing iCCM** | | | | | | | | | | | | | | | |
| Awor 2014  Uganda | | iCCM | 5 days | Frequency not stated | YES |  | YES | YES | Sub | | Free | 87.7  (CI 79.0–96.4 | 91.0  (NB low n=11) | 100  (NB low n=33) | 97.6 |
| Bagonza 2021  Uganda | | iCCM with peer supervision | 3 days for peer supervisors | Monthly |  |  |  |  |  | |  |  |  |  | 53.5-66.3^7^ |
| Kitutu 2017  Uganda | | iCCM | Duration not stated | Monthly | YES |  | Through designated wholesaler | Through designated wholesaler | Sub | | Free | 47.8 | not stated | not stated | not stated^8^ |
| Mbonye 2020  Uganda | | iCCM | 4 days | Weekly for 2 months then less frequent | YES |  | YES | YES | Not stated | | Not stated | 86.9 | 87.4 | 94.3^6^ | not stated |

^1^  approximate reading from figure

^2^  Not stated in original papers; taken from Visser et al 2017, based on Visser et al’s contact with original study authors

^3^ For Omale 2021 the indicator reported is “% febrile patients receiving RDT **from any provider**”; for O’Meara 2018 the indicator reported is “% febrile patients receiving **any malaria test from any provider**”

^4^ For Laktabai 2020, O’Meara 2016 and O’Meara 2018 the indicator reported is “% patients with negative RDT not receiving **ACT**”; For Cohen 2015 (Kenya) and O’Meara 2018 results are reported **from any provider**. For Mbonye 2015 the indicator reported is “% patients with negative RDT not receiving **AL or rectal artesunate**” and “% patients with positive test receiving **AL or rectal artesunate**”

^5^  For Modrek 2014 the indicators reported are based on medicines **taken by patients** (not on those dispensed); the indicator “% patients with negative test not receiving antimalarial” is reported only for the 57% of patients who bought both an antimalarial and drugs to treat their symptoms

^6^  For Mbonye 2020 the indicator reported is “% patients with positive test receiving **AL**”

^7^ Bagonza 2021 used interrupted time series – table includes range of results across 7 monthly post-intervention data points (no statistical difference in trend between intervention and control)

^8^ Kitutu 2017 do not report this indicator but do report an alternative indicator of appropriate treatment for malaria (see text)

^9^ Cohen 2015 Kenya results for the 11 treatment arms are not presented separately. Rather the effect of ACT and RDT subsidies is estimated by pooling arms in different combinations
