# Supplementary material for: Improving malaria case management with artemisinin-based combination therapies and malaria rapid diagnostic tests in private medicine retail outlets in sub-Saharan Africa: A systematic review
Source: PLoS One. 2024 Jul 29;19(7):e0286718. doi: 10.1371/journal.pone.0286718 (PMC11285950; doi:10.1371/journal.pone.0286718)
Supplement: Attachment — Submitted filename: Response to Editors 19May23.docx [file pone.0286718.s008.docx]

Response to Editors’ Comments - Re PONE-D-23-05583

Many thanks for your email of the 28/2/23 with comments on our manuscript. We have responded as follows *in italics*:

1. We are concerned that the literature review was completed in 2021. Systematic and scoping reviews should provide an up-to-date overview of the research topic covered. Since the literature search was completed over a year ago, we are concerned that this study does not meet this requirement.

*Many thanks for this point which we agree was justified. We have now updated all searches to 10 March 2023. As a result we have identified three additional eligible articles (Dieci et al 2023, Soniran et al 2022, Laktabai et al 2022). We have fully updated the paper to include these 3 additional articles.*

2. We are concerned that your study, while providing some data about the literature search performed, does not provide sufficient information on the methodology to meet the recommendations set out by PRISMA (Preferred Reporting Items for Systematic Reviews and Meta-Analyses).

*We were surprised by this comment as we had very carefully followed the PRISMA guidance throughout the review, and included a PRSIMA checklist with our submission detailing where each element is documented in the paper. In subsequent exchanges with the editors to query this, no specific issues were highlighted in terms of PRISMA compliance, so we have assumed that the key methodological concern related the date of the searches which has been addressed as explained above.*
