# Supplementary material for: Improving malaria case management with artemisinin-based combination therapies and malaria rapid diagnostic tests in private medicine retail outlets in sub-Saharan Africa: A systematic review
Source: PLoS One. 2024 Jul 29;19(7):e0286718. doi: 10.1371/journal.pone.0286718 (PMC11285950; doi:10.1371/journal.pone.0286718)
Supplement: Attachment — Submitted filename: My comments regarding a manuscript titled as.docx [file pone.0286718.s010.docx]

**My comments regarding a manuscript** titled as

“**Improving malaria case management with artemisinin-based combination therapies and malaria rapid diagnostic tests in private medicine retail outlets in sub-Saharan Africa: a systematic review**”

1. Given that an update version of **PRISMA 2020** is available why you prefer to use the old one **PRISMA 2015**?
2. Since your review focused on Sub-Saharan Africa but you did not include **African journal online** in your database search, why**?**
3. What are the tools you used to resolve the possible existence of biases in this review?
4. I think it is better to use a mix of figures and tables to present your results.

Overall, except the comments raised above the manuscript was well written.
