# Supplementary material for: Improving malaria case management with artemisinin-based combination therapies and malaria rapid diagnostic tests in private medicine retail outlets in sub-Saharan Africa: A systematic review
Source: PLoS One. 2024 Jul 29;19(7):e0286718. doi: 10.1371/journal.pone.0286718 (PMC11285950; doi:10.1371/journal.pone.0286718)
Supplement: Attachment — Submitted filename: Response to Reviewer 2 Comments PONE-D-23-15382.docx [file pone.0286718.s011.docx]

**Responses to reviewer comments:**

| **Comments** | **Author response** |
| --- | --- |
| **Reviewer 2** |  |
| 1. Given that an update version of PRISMA 2020 is available why you prefer to use the old one PRISMA 2015? | Thank you for pointing out this error. We did in fact follow the PRISMA 2020 guidelines (as can be seen from the PRISMA 2020 checklist submitted with this article). We have amended this in the Methods line 90. |
| 2. Since your review focused on Sub-Saharan Africa but you did not include African journal online in your database search, why? | We searched 5 databases: Medline Ovid, Embase Ovid, Global Health Ovid, Cochrane Library and Econlit Ovid, together with a wide range of grey literature sources. The 5 databases were selected for their coverage of public health, clinical medicine, health policy, infectious disease, systematic reviews and economic literature (with the latter included as several of the papers on PMR interventions are conducted by economists), and together these 5 databases provide very good coverage of articles in this field from Africa. We did not include the African Journals Online (AJOL) database, which is not one of the databases recommended for literature searching by the librarians consulted. We note that AJOL only supports a simple single field search function, which would not be appropriate for the complex search strategy applied in this review. |
| 3. What are the tools you used to resolve the possible existence of biases in this review? | We assessed the methodological quality of each paper (sometimes called the risk of bias). As stated from line 158, we chose an adjusted Downs and Black quality assessment instrument for the assessment, due to its ability to capture the wide range of study designs in this review. Minor adjustments were made to the tool to better assess the range of studies included in this review, including the addition of questions from the Effective Public Health Practice Project quality assessment tool for quantitative studies, Drummond's checklist for assessing economic evaluations, and the Newcastle-Ottawa Scale for assessing the quality of nonrandomised studies in meta-analyses. Full details on the tool and the source for each question are supplied in S2 Table. The quality assessment for each study was conducted by two authors independently. The results were used to inform the narrative synthesis, with any concerns about specific studies noted in the results. For example, we noted where results were based on studies where the study team had provided RDTs directly to the team, or where results relied on PMR’s own records (rather than independent data collection methods). |
| 4. I think it is better to use a mix of figures and tables to present your results | Thanks for this comment. We did consider the use of figures to present the results currently in Table 2a and 2b. However, given the high number of different outcomes reported across studies a figure presentation would have been very cumbersome. For readers who prefer a more visual representation of the results, the colour shading in the Heat Maps in S6 Table does provide this. |
