# Supplementary material for: Improving malaria case management with artemisinin-based combination therapies and malaria rapid diagnostic tests in private medicine retail outlets in sub-Saharan Africa: A systematic review
Source: PLoS One. 2024 Jul 29;19(7):e0286718. doi: 10.1371/journal.pone.0286718 (PMC11285950; doi:10.1371/journal.pone.0286718)
Supplement: Attachment — Submitted filename: Response to Editor and Reviewer Comments PONE-D-23-15382_v2.docx [file pone.0286718.s009.docx]

**Responses to reviewer comments:**

| **Comments** | **Author response** |
| --- | --- |
| **Reviewer 1** |  |
| 1. Why authors not used Meta-analysis for pooled estimation of the study outcomes? The final recommendation of a systematic review is qualitative while you could do it. | Our understanding is that meta-analysis should only be conducted when a group of studies is sufficiently homogeneous in terms of subjects involved, interventions, and outcomes to provide a meaningful summary. We decided a meta-analysis was not appropriate in this case because of the high degree of heterogeneity across studies in terms of their study design, outcomes, intervention and context (line 134). For example, the interventions are highly varied in terms of their focus and component parts (see S3 Table). They comprise a wide range of study designs from individual and cluster RCTs to pre-and post with and without control, ITS, and post-intervention with control. These is also considerable variation in the outcome indicators used (see Table 2a and 2b). Also see the footnotes to these tables which document the high number of variations in how outcomes in each column are calculated by different authors. Finally there is variation in the data collection tools used, which can substantially affect the degree of Hawthorne effect (see page 8 – Section on “Choice of data source reported”). We therefore concluded that a meta-analysis of any of our indicators would not lead to a meaningful summary. |
| 2. You combined various designs in the review. The design, methods, and quality assessment of each design are different. Interventional studies not only differ in design but often indicated the impact of a special intervention which is not usually performed in routine care. | It is correct that a wide range of designs were included in the review. We included randomized and non-randomized controlled trials, pre-post designs with or without a control group, and time-series or repeated measure surveys, that reported on an intervention to support the provision of RDTs and/or ACTs by PMR staff. We note that these are broader study design eligibility criteria than used in many systematic reviews. This partly reflected a desire to be as inclusive as possible of available evidence that could inform policy, but also the fact that randomised or even controlled designs may not be feasible or appropriate when evaluating large scale operational implementation of interventions such as AMFm, where distribution through standard supply chains or use of mass media severely limits the potential to restrict interventions to certain areas (see first para of Discussion). We note that to maintain rigour we excluded studies that had neither a historical nor a contemporaneous control.  We created a bespoke quality assessment checklist which was appropriate for the wide range of study designs included, and efficient to use, drawing primarily on Downs and Black, with some additions/modifications from the Effective Public Health Practice Project (EPHPP) quality assessment tool for quantitative studies, Drummond’s checklist for assessing economic evaluations, and the Newcastle-Ottawa Scale for assessing the quality of nonrandomised studies in meta-analyses. Studies with a contemporaneous control were scored out of 12, and those without, out of 9 (see S2 Table).  The reviewer comments that the studies “often indicated the impact of a special intervention which is not usually performed in routine care”. We would respond that the interventions evaluated all supported the provision of first line antimalarials and/or malaria diagnostics. These are recommended in standard treatment guidelines for the study countries. In some cases the interventions were introducing these items to PMRs (with a view to a potential wider roll out), and in others they were aiming to improve their provision in PMRs. We would consider either appropriate for inclusion in the review, and note that there is broad interest at the country and multi-lateral level in interventions such as these for improving malaria case management in PMRs (line 70-80). |
| 3. The authors reviewed malaria case management related to appropriate treatment (Artimisinib-based combination therapy) and availability of malaria diagnostic tests and RDTs, and the author mentioned “There are widespread concerns about the quality of malaria case management” However, I am surprised that authors did not use meta-analysis and reliable evidence at the Global level for providing the study rational, and also as relevant findings for presenting and comparing in the discussion:  - Availability of malaria diagnostic tests, anti-malarial drugs, and the correctness of treatment: a systematic review and meta-analysis. Malaria Journal 2023, 22(1).  - Health workers readiness and practice in malaria case detection and appropriate treatment: a meta-analysis and meta-regression. Malaria Journal 2021, 20(1). | Thank you for sharing these interesting references. We note that the studies included in these reviews are of care provided by health facilities (e.g. health centres or hospitals) or community health workers, with only one reviewed study also mentioning private medicine retailers (PMRs) (i.e. drug shops and pharmacies) though these are not separated out from other providers. Our paper focuses purely on care provided by PMRs, and the statement you mention refers specifically to PMRs i.e. “There are widespread concerns about the quality of malaria case management received from PMRs.” So we don’t feel that these specific reviews would be appropriate citations for our review. |
| 4. Authors also expressed that “As no comprehensive, up-to date systematic review was identified on strategies to improve care of febrile patients by PMR, WHO commissioned this review”. In addition to the above paper please see the below article: Development and validation of an online tool for assessment of health care providers’ management of suspected malaria in an area, where transmission has been interrupted. Malar J 21, 304 (2022). I think that “no comprehensive” should be replace by “limited evidence” | Please note that our statement “As no comprehensive, up-to date systematic review was identified on strategies to improve care of febrile patients by PMR, WHO commissioned this review” refers to systematic literature reviews specifically on strategies to improve care provided by private medicine retailers (PMR). None of the papers you suggest are of this type and scope. We have therefore left the text unchanged. |
| 5. Why you had no search on Scopus and ISI? | We searched 5 databases: Medline Ovid, Embase Ovid, Global Health Ovid, Cochrane Library and Econlit Ovid, together with a wide range of grey literature sources. The 5 databases were selected for their coverage of public health, clinical medicine, health policy, infectious disease, systematic reviews and economic literature (with the latter included as several of the papers on PMR interventions are conducted by economists). We also considered Scopus which covers some social science not covered by Econlit but decided not to include it given that quantitative intervention evaluations eligible for inclusion in this study would be highly likely to be included in at least one of Medline, Embase or Econlit. We did not consider ISI, which is not one of the databases recommended for literature searching by the library of the London School of Hygiene and Tropical Medicine. |
| 6. Please mention exact PubMed used search strategy in the text | Please not that we did not search PubMed, but Medline Ovid (which has very similar but slightly broader coverage). We describe the search domains used line 117-118, but have placed the exact search strategy in the Supplementary materials S1 Table for all the databases, due to the level of detail involved. |
| 7. Where did you search unpublished records? | As stated line 131-2, unpublished studies were identified by contacting researchers and practitioners in the field. These included Abigail Pratt, Charles E. Okafor, Daniel Rosen, James Tibenderana, Jessica Cohen, Justin Cohen, Prashant Yadav, Phyllis Awor, Rachel Silverman, Romuald Mbwasi, Sian Clarke, Stefan Peterson, Stephen Poyer, Ugwu Omale, Wendy O'Meara, and Elizabeth Streat. We also checked the bibliographies of published studies. |
| **Editor comments** |  |
| 8. Please ensure that your manuscript meets PLOS ONE's style requirements, including those for file naming | Files have been named as per guidance. |
| 9. Thank you for stating the following in your Competing Interests section: "No authors have competing interests"  Please complete your Competing Interests on the online submission form to state any Competing Interests. If you have no competing interests, please state "The authors have declared that no competing interests exist.", as detailed online in our guide for authors at http://journals.plos.org/plosone/s/submit-now  This information should be included in your cover letter; we will change the online submission form on your behalf. | This statement has been included in our cover letter. |
| 10. We note that you have included the phrase “data not presented” in your manuscript. Unfortunately, this does not meet our data sharing requirements. PLOS does not permit references to inaccessible data. We require that authors provide all relevant data within the paper, Supporting Information files, or in an acceptable, public repository. Please add a citation to support this phrase or upload the data that corresponds with these findings to a stable repository (such as Figshare or Dryad) and provide and URLs, DOIs, or accession numbers that may be used to access these data. Or, if the data are not a core part of the research being presented in your study, we ask that you remove the phrase that refers to these data. | On lines 423 and 449-450 we have removed the words “data not presented in Tables”. Citations were already included here to support these statements.  On line 390-391 we have clarified that we are referring to the data the original authors of the reviewed paper provided. This now says “data not provided by authors for PMR clients only”. |
